# Pyrvinium Attenuates Amiodarone-Induced Pulmonary Fibrosis in Male Rats: Association with Modulation of Wnt/β-Catenin Related Proteins

**DOI:** 10.3390/pharmaceutics18101289

**Published:** 2026-10-10

**Authors:** Shaima Kutbi, Hala Attia, Nouf M. Alrasheed, Maha M. Arafah, Ahlam M. Alhusaini, Maha A. Al-Amin, Rehab Ali, Wedad S. Sarawi

**Affiliations:** 1 Department of Pharmacology and Toxicology, College of Pharmacy, King Saud University, P.O. Box 2455, Riyadh 11451, Saudi Arabia; 443203789@student.ksu.edu.sa (S.K.); nrasheed@ksu.edu.sa (N.M.A.); aelhusaini@ksu.edu.sa (A.M.A.); mahaali@ksu.edu.sa (M.A.A.-A.); reali@ksu.edu.sa (R.A.); wsarawi@ksu.edu.sa (W.S.S.); 2 Pathology Department, College of Medicine, King Saud University, P.O. Box 2457, Riyadh 11451, Saudi Arabia; marafah@ksu.edu.sa

**Keywords:** amiodarone, lung fibrosis, Wnt/β-catenin, pyrvinium

## Abstract

**Background/Objectives:** Amiodarone (AmoD) is an effective antiarrhythmic drug whose use is limited by life-threatening pulmonary fibrosis. Although Wnt/β-catenin signaling contributes to several lung diseases, its involvement in AmoD-induced pulmonary fibrosis remains unknown. This study investigated whether Wnt/β-catenin signaling is altered during AmoD-induced pulmonary fibrosis and whether its pharmacological modulation with pyrvinium (Pyrv) attenuates this toxicity. **Methods:** Rats were allocated into four groups and treated for five weeks as follows: normal control, AmoD (30 mg/kg/day), Pyrv (60 µg/kg/day), and AmoD + Pyrv. Pulmonary injury and fibrosis were evaluated using histopathological examination, Masson’s trichrome staining, immunohistochemical assessment of collagen I, α-smooth muscle actin (α-SMA), and transforming growth factor-β1 (TGF-β1), and measurement of inflammatory cytokines, tumor necrosis factor-α (TNF-α) and interleukin-6 (IL-6), and Th1/Th2-related cytokines. Wnt/β-catenin-associated proteins, including Wnt3a, active β-catenin, and glycogen synthase kinase-3β (GSK-3β), were evaluated by Western blotting. **Results:** AmoD induced marked pulmonary architectural disruption, inflammatory cell infiltration, increased lung weight/body weight ratio, collagen deposition, and increased expression of TGF-β1, α-SMA, and collagen I, as well as increased levels of TNF-α, IL-6, and disruption of Th1/Th2 immune balance. At the molecular level, AmoD increased Wnt3a, active β-catenin, and the p-GSK-3β/total GSK-3β ratio. Co-administration of Pyrv significantly attenuated the histopathological, inflammatory, immune, and fibrotic alterations and reduced the AmoD-associated changes in Wnt/β-catenin-related proteins. **Conclusions:** The findings indicate that AmoD-induced pulmonary injury and fibrosis are associated with modulation of Wnt/β-catenin related proteins and that Pyrv treatment attenuates these pathological changes. Targeting this pathway could be a promising protective strategy to prevent fibrogenesis during AmoD treatment.

## 1. Introduction

Amiodarone (AmoD), a class III anti-arrhythmic agent, was first approved in 1985 by the U.S. FDA for the management of potentially life-threatening ventricular arrhythmias [1]. Despite its therapeutic efficacy, long-term use of AmoD is associated with serious adverse effects involving multiple organ systems. Among these, pulmonary toxicity represents the most severe and life-threatening complication, occurring in approximately 5–15% of treated patients, with a mortality rate ranging between 5% and 10% [2,3].

AmoD-induced lung toxicity typically manifests as interstitial pneumonitis that progresses to pulmonary fibrosis, ultimately causing irreversible respiratory failure [4]. The underlying pathogenesis is multifactorial and involves direct cytotoxicity [5], oxidative stress, inflammatory response, mitochondrial dysfunction, immune dysregulation, and intracellular phospholipid accumulation (phospholipidosis) [6,7]. In addition, AmoD may activate the renin-angiotensin system, which triggers pulmonary injury through enhancement of oxidative stress and induction of profibrotic mediators, including transforming growth factor-β1 (TGF-β1) [8]. Alteration in pulmonary immune response also plays a major role, characterized by a pathognomonic shift toward a T helper 2 (Th2) response (elevated Th2-derived interleukin-4 [IL-4] alongside suppression of Th1-derived interferon-γ [IFN-γ]), which drives fibroblast activation and extracellular matrix (ECM) accumulation [8]. Despite these observations, the molecular pathways contributing to AmoD-induced lung fibrosis remain unclear and require further investigation.

The Wingless/integrase-1 (Wnt) signaling pathway is a conserved signaling network involved in tissue development, repair, stem cell maintenance, and organ homeostasis [9]. Wnt signaling, which relies on the transcriptional coactivator β-catenin, is classified as the canonical Wnt pathway [10] and orchestrates nuclear transcription of genes controlling cellular proliferation, differentiation, and migration [11]. In the absence of Wnt stimulation, cytosolic β-catenin is constitutively degraded by a destruction complex that includes glycogen synthase kinase-3β (GSK-3β), the key kinase responsible for β-catenin phosphorylation and targeted proteasomal breakdown. Canonical pathway activation occurs when specific Wnt ligands, such as Wnt3a, bind to Frizzled receptors and low-density lipoprotein receptor-related protein 5/6 (LRP5/6) co-receptors. This interaction inactivates the destruction complex, thereby promoting the stabilization, accumulation, and translocation of non-phosphorylated (active) β-catenin into the nucleus, where it regulates transcription of genes involved in cellular proliferation, differentiation, tissue remodeling, and ECM regulation [11].

Increasing evidence indicates that dysregulated Wnt/β-catenin signaling is associated with pulmonary fibrotic diseases [12,13,14,15,16,17]. Following lung injury, aberrant activation of this pathway was indicated, as evidenced by β-catenin accumulation in fibrotic lung tissue [14]. Wnt/β-catenin signaling also acts synergistically with TGF-β1, contributing to persistent ECM remodeling [10,13,14,15]. In experimental models of bleomycin-induced pulmonary fibrosis, elevated β-catenin expression strongly correlates with increased profibrotic cytokines, upregulation of mesenchymal markers such as α-smooth muscle actin (α-SMA) and collagen I, thereby contributing to lung damage [14,16,17]. These observations suggest that Wnt/β-catenin signaling may also participate in pulmonary fibrosis induced by toxicological agents such as AmoD and could be a promising target to prevent fibrosis and preserve lung integrity during AmoD treatment.

Pyrvinium (Pyrv) is an FDA-approved anthelmintic compound that has been investigated experimentally as an antagonist of canonical Wnt/β-catenin signaling via enhancing the casein kinase 1α-dependent β-catenin degradation [18]. Pyrv has demonstrated protective effects in several experimental models involving fibrosis, inflammation, and tissue injury [11,18,19,20]. However, its effects against AmoD-induced lung injury and fibrosis have not been studied.

To date, the role of Wnt/β-catenin in the pathogenesis of AmoD-induced pulmonary fibrosis remains unknown and has not been explored. Therefore, this study aimed to investigate whether canonical Wnt/β-catenin signaling is altered in an experimental model of AmoD-induced pulmonary fibrosis and to examine whether pharmacological modulation of this pathway with Pyrv could protect against such toxicity by alleviating pulmonary injury, inflammation, immune dysregulation, and fibrotic remodeling in male rats.

## 2. Materials and Methods

### 2.1. Materials

Amiodarone hydrochloride (Cordarone^®^, 200 mg tablets; Sanofi, France) was purchased from a local pharmacy in Riyadh, Saudi Arabia. Pyrvinium pamoate bpowder (Cat. No. IP3860) and rat enzyme-linked immunosorbent assay (ELISA) kits for determining tumor necrosis factor-α (TNF-α, Cat. No. SEKR-0009) and IL-6 (Cat. No. SEKR-0005) were purchased from Solarbio Life Sciences (Beijing, China). Rat ELISA kits for measuring IL-4 (Cat. No. MBS700367) and IFN-γ (Cat. No. MBS2700898) were purchased from MyBiosource (San Diego, CA, USA). Primary antibodies against α-SMA (Cat. No. A7248) and GSK-3β (Cat. No. A2081) were obtained from ABclonal (Woburn, MA, USA). Primary antibodies against active (non-phosphorylated) β-catenin (Cat. No. 8814) and phosphorylated GSK-3β (p-GSK-3β, Ser9, Cat. No. 9336) were purchased from Cell Signaling Technology (Danvers, MA, USA). Anti-TGF-β1 antibody (Cat. No. FNab08638) was obtained from FineTest (Wuhan, China), while antibodies against Wnt3a (Cat. No. K000174M) and collagen I (Cat. No. K002759P) were purchased from Solarbio Life Sciences (Beijing, China).

### 2.2. Animals

Twenty-four male Wistar albino rats, aged 6 to 8 weeks and weighing 190–250 g were obtained from the Animal Care Center, College of Pharmacy, King Saud University, Riyadh, Saudi Arabia. Animals were acclimatized for one week prior to experimental procedures and maintained under controlled laboratory conditions (temperature: 25 ± 2 °C, relative humidity: 55–60%, 12-h light/dark cycle) with unlimited access to standard diet pellets and water. Only male rats were utilized to minimize hormonal variability associated with the estrous cycle, ensuring consistent assessment of AmoD-induced pulmonary fibrosis and the effects of Pyrv. All experiments were conducted in accordance with the ethical guidelines of King Saud University and were approved by the institutional Research Ethics Committees (Approval No. KSU-SE-24-48) on (13/10/2024). All histopathological evaluation, immunohistochemical analysis, and ImageJ-based quantification (version 1.35k) were conducted by blinded investigators using coded samples. The study protocol follows the ARRIVE 2.0 guidelines [21].

### 2.3. Experimental Design

Following the acclimatization period, rats were randomly assigned to four experimental groups using a computer-generated simple randomization procedure to minimize allocation bias (*n* = 6 per group). Rats were then treated for five consecutive weeks as follows: Group I (normal control group): rats were given an equivalent volume of 0.9% saline (the vehicle of AmoD) by oral gavage; Group II (Pyrv): rats were treated with Pyrv (60 μg/kg/day) [18,20] by oral gavage; Group III (AmoD): rats received AmoD (30 mg/kg/day) by oral gavage [8,22]; Group IV (AmoD + Pyrv): rats were treated with Pyrv (60 μg/kg/day) concomitantly with AmoD (30 mg/kg/day) by oral gavage.

Pyrv powder was dissolved in dimethyl sulfoxide (DMSO) to prepare a 10 mg/mL stock solution. Prior to daily administration, the stock solution was diluted with sterile saline to achieve the target dose while maintaining a final DMSO concentration of 0.3% (*v*/*v*) (<1%). Pyrv was administered via oral gavage at a dose of 60 μg/kg/day in a fixed administration volume of 2 mL/kg.

The dose of Pyrv was selected based on previous studies employing Pyrv as a pharmacological antagonist of the Wnt/β-catenin pathway in different rat models, including isoproterenol-induced cardiotoxicity [18], renal fibrosis associated with hypertension [20], and polymicrobial sepsis [23]. AmoD tablets were finely crushed and suspended in sterile 0.9% saline. A stock suspension was prepared by dispersing 400 mg (two 200 mg tablets) of amiodarone hydrochloride in 26.6 mL of 0.9% saline to yield a final concentration of approximately 15 mg/mL. The suspension was homogenized thoroughly prior to each draw, and oral gavage was administered at a volume of 2 mL/kg to deliver the target dose of 30 mg/kg/day [8,22]. Control animals received an equivalent volume (2 mL/kg) of sterile 0.9% saline.

### 2.4. Sample Collection and Preparation of Lung Homogenate

Twenty-four hours following the final treatment, animal body weights were recorded. Rats were humanely euthanized using carbon dioxide displacement at a gradual chamber volume rate of 10–30% per minute to minimize distress prior to loss of consciousness, followed immediately by decapitation. Lungs were rapidly excised, rinsed in ice-cold phosphate-buffered saline (PBS, pH 7.4), and weighed to determine the lung index [lung weight/body weight (LW/BW) ratio]. The right lung tissue was homogenized in ice-cold PBS (1:10 *w*/*v*) and centrifuged at 5000× *g* for 5 min at 4 °C. The resulting supernatant was collected and stored at −80 °C for subsequent biochemical and ELISA analyses. The upper lobe of the left lung was fixed in 10% neutral buffered formalin for 24 h for histological and immunohistochemical evaluations, while the remaining lung tissue was snap-frozen in liquid nitrogen and stored at −80 °C for immunoblotting.

### 2.5. Calculation of Lung Index

The lung index was calculated as the ratio of lung weight (LW, measured in mg) to body weight (BW, measured in g).

### 2.6. Enzyme-Linked Immunosorbent Assay (ELISA)

Concentrations of pro-inflammatory cytokines (TNF-α, IL-6) and Th2 and Th1-associated cytokines (IL-4, IFN-γ) were quantified in lung tissue homogenates using rat-specific sandwich ELISA kits according to the manufacturers’ protocols. All standards and biological samples were assayed in duplicate, and optical density was measured at 450 nm using a microplate spectrophotometer. Cytokine concentrations were derived from standard curves and normalized to tissue wet weight, expressed as pg/g tissue.

### 2.7. Histological Examination

Formalin-fixed lung tissues were dehydrated through a graded ethanol series, cleared in xylene, and embedded in paraffin blocks. Sections of 5 μm thickness were cut using a rotary microtome (Leica Biosystems, Nussloch, Germany). Sections were stained with Hematoxylin and Eosin (H&E) for assessment of pulmonary architecture, pathological changes and inflammatory infiltration, and with Masson’s trichrome for visualization of connective tissue and collagen deposition (fibrosis). Lung sections were subjected to blinded histopathological scoring, which focused on alveolar wall thickening, hemorrhage, edema, inflammatory infiltration and fibrosis according to the following score: 0, no injury; 1, <25% injury; 2, 25–50%; 3, 50–75%; and 4, >75% [24]. In addition, fibrosis scoring was performed according to Ashcroft’s protocol [25]. Ashcroft Scoring includes 8 criteria (0–8 scale): 0 = Normal tissue, no fibrosis, 1 = Minimal fibrous thickening of alveolar/basement membranes, 2 = Mild fibrotic changes, no obvious disruption of architecture, 3 = Moderate fibrosis with distinct thickening of septal walls, 4 = Moderate-to-severe fibrosis with architectural distortion, 5 = Severe fibrosis with obvious disruption of normal structure, 6 = Extensive fibrosis with marked architectural destruction, 7 = Severe fibrosis with “honeycomb” appearance (lung) or complete distortion, 8 = Total fibrous obliteration of normal tissue architecture. Slides were examined under a light microscope (Olympus BX53, Tokyo, Japan). Lung injury scoring, Ashcroft scoring, and quantification of collagen accumulation were performed on one section per animal, and three non-overlapping microscopic fields were analyzed per section using ImageJ software (version 1.35k; NIH, Bethesda, MD, USA) for a total of 18 fields per group, at magnification 200X, in the anatomical middle zone. Field-level measurements were averaged to generate a single value for each animal before statistical analysis, confirming that the individual rat, and not individual microscopic fields, served as the primary experimental unit for all statistical analyses.

### 2.8. Immunohistochemistry

Paraffin-embedded lung sections of 5 μm thickness were deparaffinized in xylene and rehydrated through a descending alcohol series. Antigen retrieval was performed by heating the sections in 10 mM sodium citrate buffer (pH 6.0) at 95 °C for 10 min. Endogenous peroxidase activity was blocked with 3% hydrogen peroxide for 10 min, followed by blocking of non-specific binding with 5% normal goat serum for one hour at room temperature. Sections were incubated overnight at 4 °C with primary antibodies against collagen I (1:200), α-SMA (1:300), and TGF-β1 (1:200). After washing with PBS, sections were incubated with biotinylated secondary antibody (Vector Laboratories, Burlingame, CA, USA) for two hours, visualized using 3,3′-diaminobenzidine (DAB) substrate, and counterstained with Mayer’s hematoxylin. Immunopositive area fraction (%) and mean optical density were quantified using ImageJ software (version 1.35k). Quantification was performed on one section per animal, and three non-overlapping microscopic fields were analyzed per section using ImageJ software (version 1.35k; NIH, Bethesda, MD, USA), for a total of 18 fields per group, at magnification 200X, in the anatomical middle zone. Field-level measurements were averaged to generate a single value for each animal before statistical analysis; therefore, the individual rat was served as the primary experimental unit for all statistical analyses.

### 2.9. Western Immunoblotting

Portions of frozen lung tissue (*n* = 3, three biological replicates per group) were homogenized in radioimmunoprecipitation assay (RIPA) lysis buffer (UFC Bio, Niagara Falls, NY, USA) supplemented with a 1% protease and phosphatase inhibitor cocktail (Sigma-Aldrich, Darmstadt, Germany). Total protein concentrations were determined using the bicinchoninic acid (BCA) protein assay kit (Santa Cruz Biotechnology, Dallas, TX, USA). Equal amounts of protein (30 μg/lane) were separated by 12.5% sodium dodecyl sulfate-polyacrylamide gel electrophoresis (SDS-PAGE) and electrotransferred onto polyvinylidene difluoride (PVDF) membranes using standard protocols. Membranes were blocked with EveryBlot Blocking Buffer (Bio-Rad Laboratories, Hercules, CA, USA) for 10 min at room temperature and incubated overnight at 4 °C with the following primary antibodies: anti-Wnt3a (1:1000), anti-active non-phosphorylated β-catenin (1:1000), anti-GSK-3β (1:1000), anti-p-GSK-3β (1:1000), and anti-GAPDH (1:3000; internal loading control). Following washes, membranes were incubated with horseradish peroxidase (HRP)-conjugated secondary antibodies (1:5000; Invitrogen, Carlsbad, CA, USA) for 1 h at room temperature. Immunoreactive protein bands were visualized using enhanced chemiluminescence (ECL; Thermo Fisher Scientific, Waltham, MA, USA) on a ChemiDoc Touch Imaging System (Bio-Rad), and signal densitometry was quantified using ImageJ software (version 1.35k; NIH, Bethesda, MD, USA). Densitometric values for each target protein band were normalized to their corresponding GAPDH loading control band from the same gel. For phosphorylated GSK-β, both p-GSK-3β and total GSK-3β band intensities were first normalized independently to GAPDH (p-GSK-3β/GAPDH and GSK-3β/GAPDH). The relative phosphorylation ratio was subsequently calculated by dividing the normalized p-GSK-3β value by the normalized total GSK-3β value.

### 2.10. Statistical Analysis

Data are presented as mean ± standard error of the mean (SEM). Statistical comparisons among experimental groups were performed using one-way analysis of variance (ANOVA) followed by Tukey–Kramer multiple comparison test using GraphPad Prism 9.3.0 software (San Diego, CA, USA). Data normality and homogeneity of variance were evaluated using the Shapiro–Wilk test. A *p*-value < 0.05 was considered statistically significant. No animals died, reached predefined humane endpoints prematurely, or were excluded during the study. All randomized animals (*n* = 6/group) successfully completed the experimental protocol, and 100% of collected samples/data were included in the final statistical analyses without exclusion. Statistical data for ELISA, Western Blot, and histological/immunohistochemical quantifications are provided in the Supplementary Material S1.

## 3. Results

### 3.1. Pyrv Ameliorates AmoD-Induced Histopathological Changes and Pulmonary Injury

**Figure 1:**
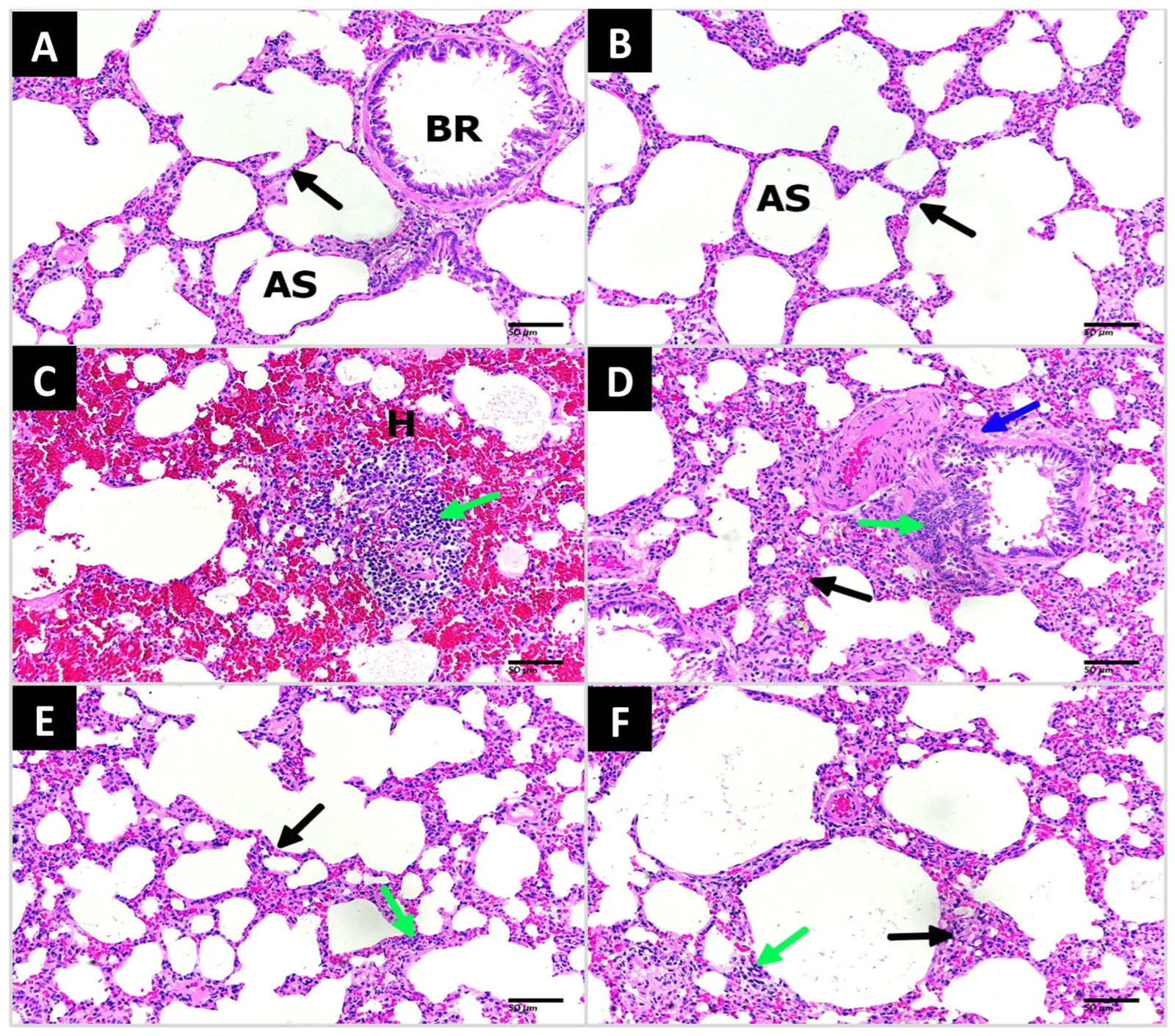
Microscopic images of lung tissue stained with hematoxylin and eosin (200X; scale bar = 50 μm). (**A**) and (**B**) Lung sections from the normal control and pyrvinium (Pyrv) groups, respectively, showing normal lung architecture with thin alveolar septa and well-opened alveolar sacs (AS) and bronchioles (BR). (**C**,**D**) Lung sections from amiodarone (AmoD)-intoxicated rats exhibiting severe lung damage, including extensive hemorrhage (H), massive inflammatory cell infiltration (green arrows), thickened interalveolar septa (black arrows), and formation of hyaline membranes (blue arrows). (**E**,**F**) Lung sections from the AmoD + Pyrv group showed marked improvement in lung architecture with reduced inflammatory infiltration, decreased septal thickening and reduced tissue injury compared with the AmoD group.

H&E staining was performed to evaluate pulmonary architecture, inflammatory cell infiltration, and structural tissue injury across the experimental groups (Figure 1). The normal control group displayed normal lung architecture characterized by thin interalveolar septa, well-expanded alveolar spaces, and intact bronchiolar epithelium (Panel A). Similarly, rats treated with Pyrv alone exhibited normal, preserved pulmonary architecture without pathological alterations (Panel B). Conversely, the AmoD-treated group developed severe histopathological lesions, including extensive intra-alveolar hemorrhage, dense inflammatory cell infiltration, marked interalveolar septal thickening, and hyaline membrane formation (Panels C and D). Co-treatment of Pyrv with AmoD noticeably attenuated these pathological changes, with reduced alveolar hemorrhage, diminished inflammatory cell infiltration, and thinner interalveolar septa compared with the AmoD group (Panels E and F). The lung injury pathological score (Table 1) showed that the normal control and Pyrv control groups exhibited normal pulmonary structure (total score = 0) in both groups. In contrast, the AmoD-treated group showed marked lung injury, reflected by the highest pathological score (mean total score = 18.6 ± 0.09). Co-administration of Pyrv with AmoD substantially reduced these alterations. The AmoD + Pyrv group displayed moderate hemorrhage and edema, with reduced inflammatory infiltration and less alveolar wall thickening, while fibrosis was limited to a minimal level (mean total score = 10.5 ± 0.2) (Table 1). Collectively, these findings indicate Pyrv’s protective effect and its ability to attenuate lung injury associated with AmoD exposure.

### 3.2. Pyrv Attenuates AmoD-Induced Pulmonary Inflammation

**Figure 2:**
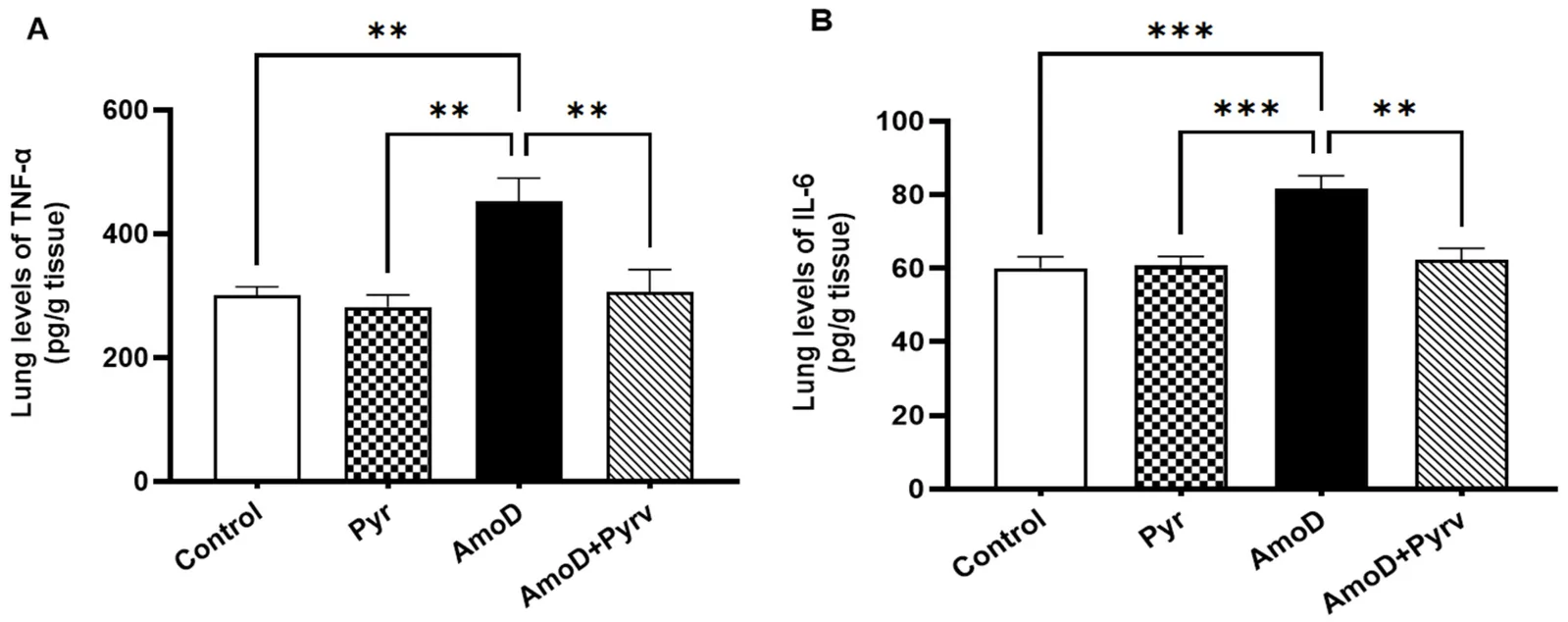
Effect of pyrvinium (Pyrv) on pro-inflammatory cytokines in amiodarone (AmoD)-induced lung injury. (**A**) Tumor necrosis factor-α (TNF-α) and (**B**) interleukin-6 (IL-6) levels. Data are presented as mean ± SEM (*n* = 6). Statistical analysis was performed using one-way ANOVA followed by Tukey–Kramer multiple comparison test. ** *p* ˂ 0.01 and *** *p* ˂ 0.001.

To assess whether Pyrv modulates the inflammatory response associated with AmoD, concentrations of TNF-α and IL-6 were quantified in lung tissue homogenates (Figure 2). AmoD administration significantly increased lung TNF-α (*p* = 0.0063) and IL-6 (*p* = 0.0005) levels compared with the normal control group. Co-administration of Pyrv significantly reduced lung TNF-α and IL-6 levels compared with the AmoD group (*p* = 0.0085 and *p* = 0.0016, respectively). These findings demonstrate an anti-inflammatory effect of Pyrv in AmoD-induced pulmonary injury and are consistent with the suggestion that modulation of Wnt/β-catenin signaling contributes to this response.

### 3.3. Pyrv Attenuates AmoD-Induced Increases in Lung Index and Pulmonary Fibrosis

#### 3.3.1. Pyrv Reduces the Lung Weight-to-Body Weight (LW/BW) Ratio

**Figure 3:**
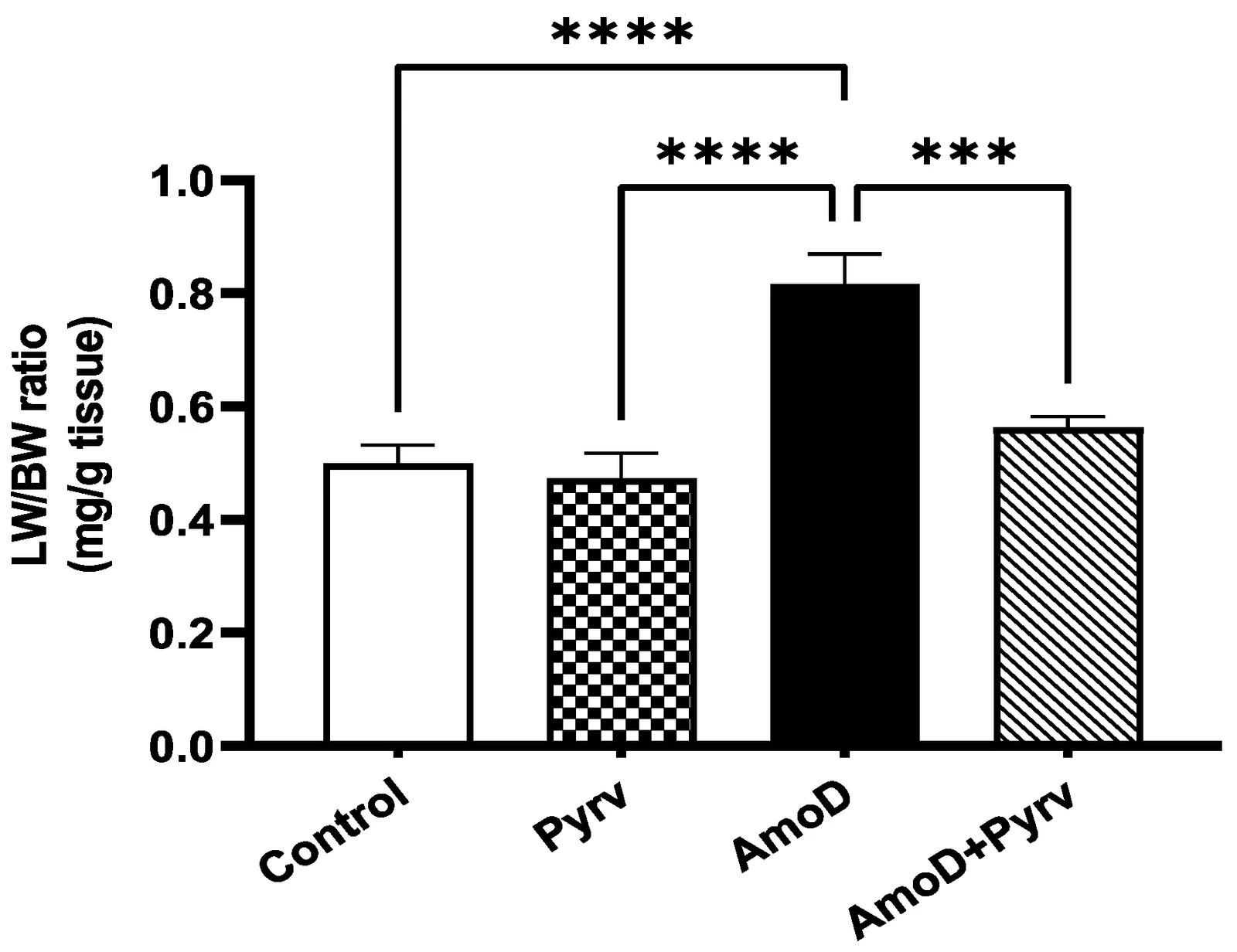
Effect of pyrvinium (Pyrv) on lung weight to body weight (LW/BW) ratio in amiodarone (AmoD)-induced lung injury. Data are presented as mean ± SEM (*n* = 6). Statistical analysis was performed using one-way ANOVA followed by Tukey–Kramer multiple comparison test. *** *p* < 0.001 and **** *p* < 0.0001.

Five weeks of AmoD administration significantly increased the LW/BW ratio compared with the normal control group (*p* < 0.0001; Figure 3). This increase may reflect pathological changes associated with lung injury, including edema, hemorrhage, inflammatory infiltration, and fibrotic tissue accumulation. Co-treatment with Pyrv significantly reduced the LW/BW ratio compared with the AmoD group (*p* = 0.0005), restoring values toward those observed in the control group (Figure 3). These findings suggest that Pyrv attenuates AmoD-induced pulmonary tissue alterations associated with increased relative lung weight.

#### 3.3.2. Pyrv Reduced the Collagen Deposition in Lung Tissue

**Figure 4:**
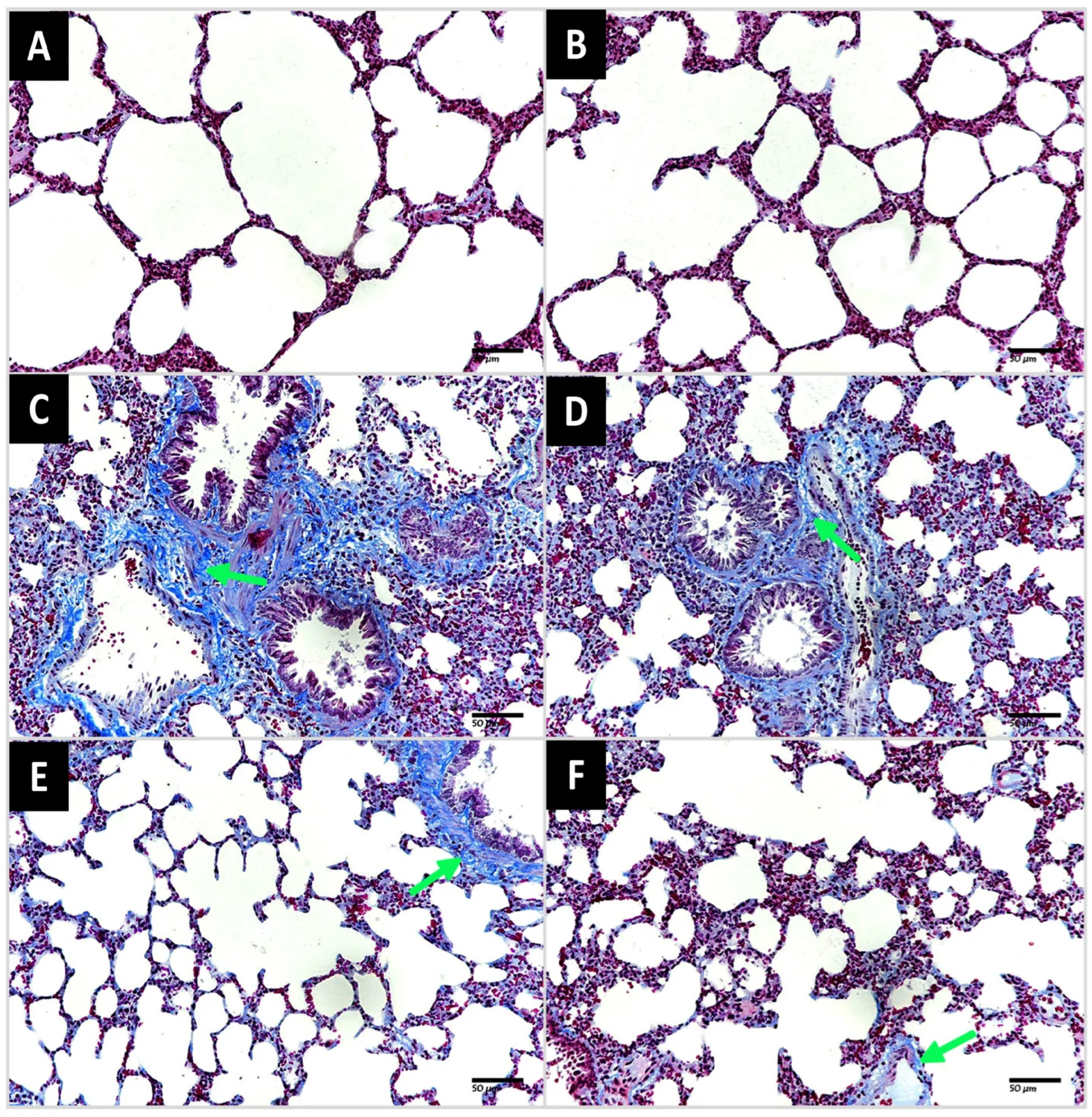

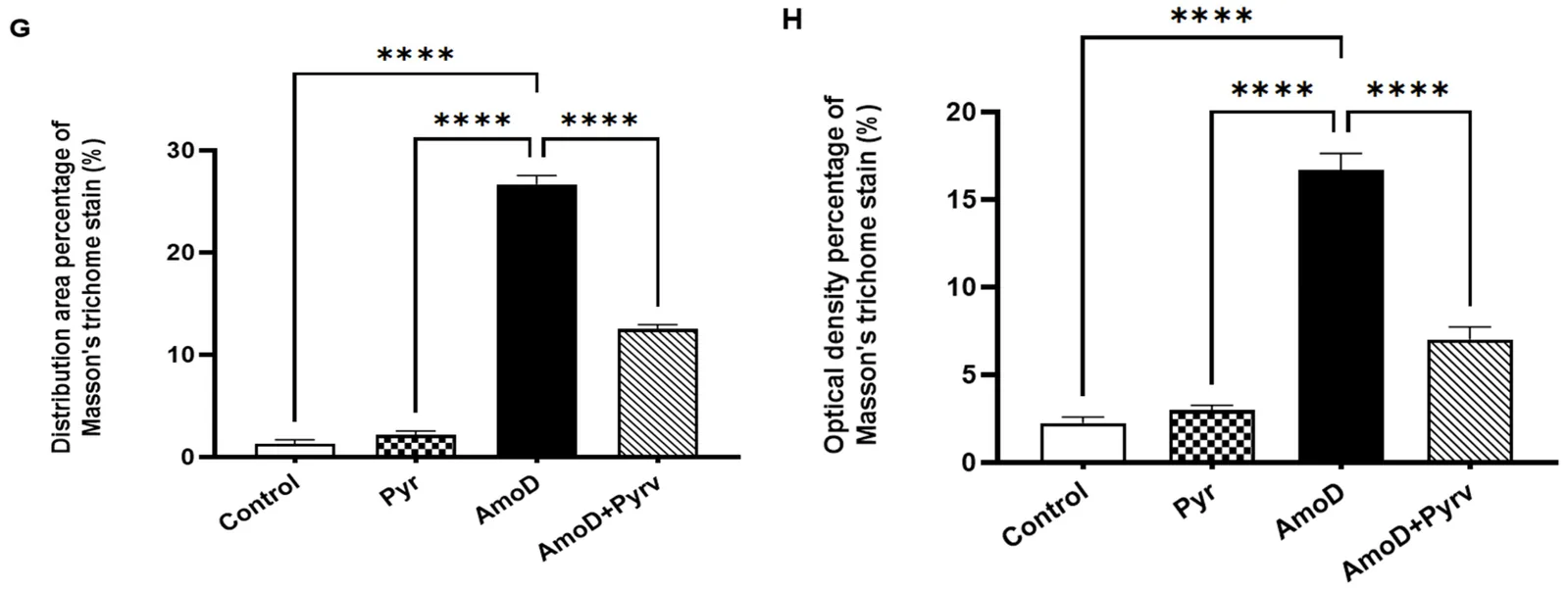
Representative photomicrographs of Masson’s trichrome-stained lung sections (200X scale bar = 50 μm). (**A**,**B**) Lung sections from normal control and pyrvinium (Pyrv) control groups, respectively, showing normal collagen distribution mainly confined to perivascular and peribronchial regions. (**C**,**D**) Lung sections from amiodarone (AmoD)-intoxicated rats displaying extensive and diffuse collagen deposition throughout the lung parenchyma, particularly around alveolar septa, with formation of dense fibrotic foci (green arrows). (**E**,**F**) Sections from AmoD + Pyrv group showing a marked reduction in collagen deposition and attenuation of fibrotic foci (green arrows). Quantitative assessment of fibrosis: (**G**) collagen area percentage and (**H**) optical density percentage of Masson’s trichrome-positive staining. The AmoD group showed a significant increase in fibrotic content compared to controls (*p* < 0.0001), whereas Pyrv co-treatment significantly reduced these levels. Data are presented as mean ± SEM (*n* = 6 rats). Statistical analysis was conducted using one-way ANOVA followed by Tukey–Kramer multiple comparison test. **** *p* < 0.0001.

Masson’s trichrome staining was used to assess collagen accumulation in lung tissue (Figure 4). The normal control and Pyrv control groups (Panels A & B, respectively) showed normal collagen distribution, primarily in expected perivascular and peribronchial spaces. In contrast, the AmoD group (Panels C and D) demonstrated extensive, diffuse collagen deposition throughout the lung parenchyma, particularly around the alveolar septa, with the formation of dense fibrotic foci. Co-treatment with Pyrv resulted in a substantial reduction in the extent of collagen deposition and fibrotic areas (Panels E and F) compared with the AmoD group. Quantitative analysis confirmed a significant increase in collagen-positive area (Panel G, *p* < 0.0001) and optical density (Panel H, *p* < 0.0001) in the AmoD-treated group compared with the normal control group. Notably, Pyrv co-administration significantly reduced both measures compared with AmoD treatment (*p* < 0.0001). These findings indicate that Pyrv attenuated the collagen accumulation associated with AmoD-induced pulmonary fibrotic remodeling.

#### 3.3.3. Pyrv Improved the Ashcroft Lung Fibrosis Scoring (Table 2)

In Ashcroft’s lung fibrosis score, the AmoD-intoxicated group showed a significant increase in score (3.6 ± 0.2) compared to the control, which had a score of zero (*p* < 0.0001). This score of a relative 4 indicates moderate-to-severe fibrosis with architectural distortion according to Ashcroft grading. AmoD + Pyrv group showed a statistically significant reduction in the score compared to the AmoD group (2.3 ± 0.16 Vs. 3.6 ± 0.2, *p* < 0.0001).

**Table 2:** Effect of Pyrv on Ashcroft Lung Fibrosis Scoring in AmoD-induced lung fibrosis.

Groups	Ashcroft Lung Fibrosis Scoring
Normal Control	0
Pyrv	0
AmoD	3.6 ^a^**** ± 0.2
AmoD + Pyrv	2.3 ^b^**** ± 0.16

Data are presented as mean ± SEM (*n* = 6 rats). Statistical analysis was conducted using one-way ANOVA followed by Tukey–Kramer multiple comparison test. a: significance versus the normal control group. b: Significance versus AmoD. **** *p* < 0.0001.

#### 3.3.4. Pyrv Ameliorates the AmoD-Induced Overexpression of Profibrotic Markers (Collagen I, α-SMA, and TGF-β1) in Lung Tissue

**Figure 5:**
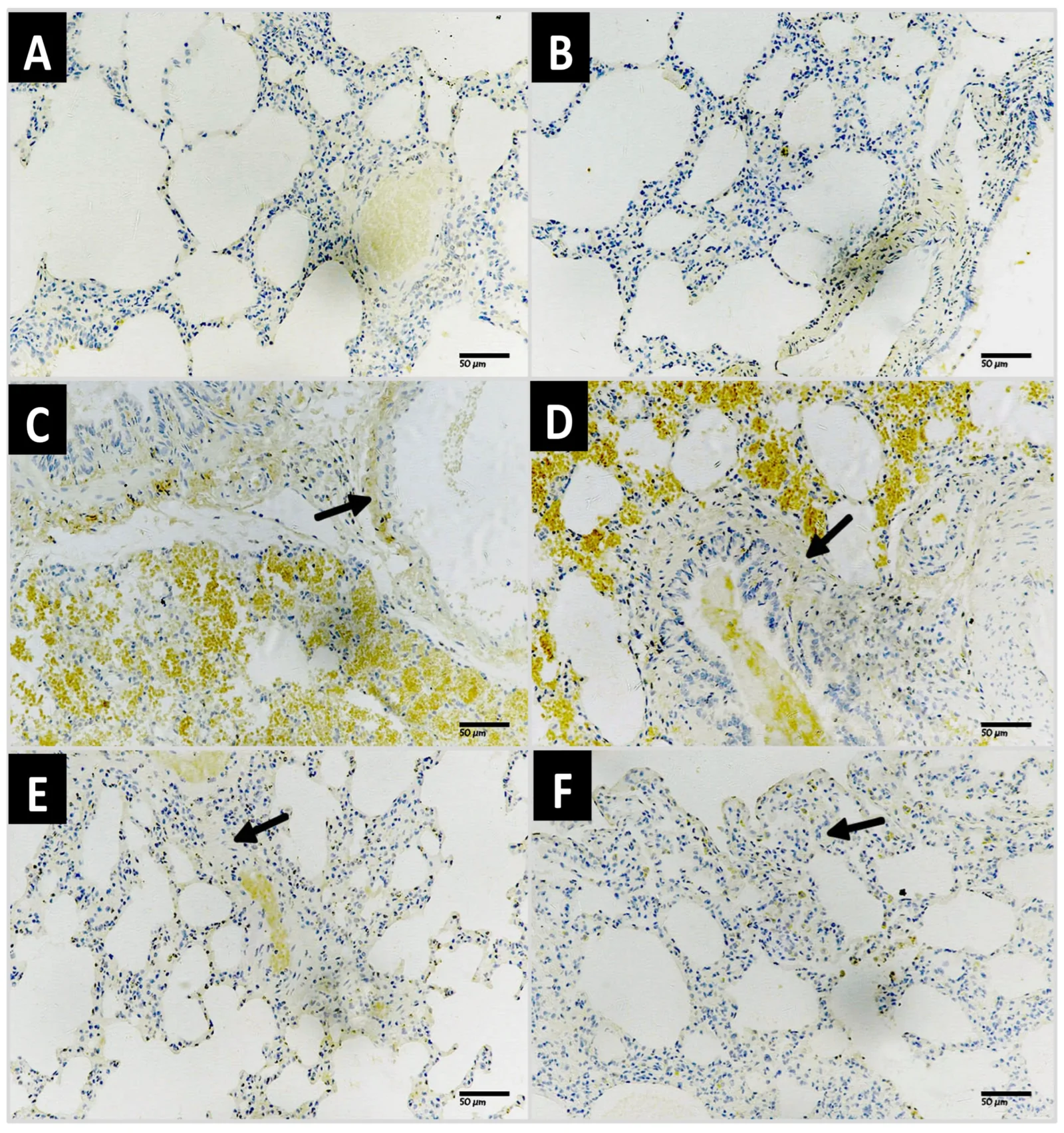

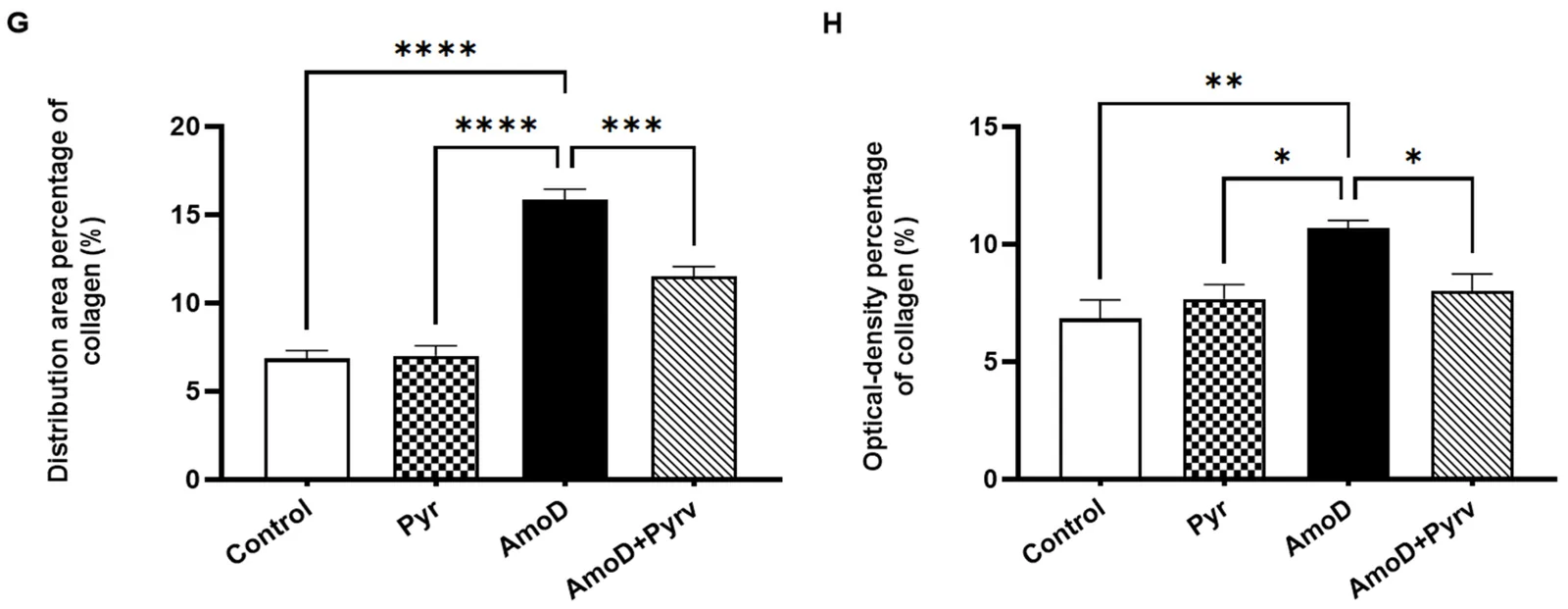
Representative photomicrographs of lung tissue immunohistochemically stained for collagen type I, showing expression in alveolar walls and interalveolar septa (200X; scale bar = 50 μm). (**A**) and (**B**) Lung sections from normal control and pyrvinium (Pyrv) control groups, respectively showing weak immunoreactivity for collagen Type I. (**C**,**D**) Lung sections from amiodarone (AmoD)-intoxicated rats exhibiting marked upregulation of collagen expression (black arrows). (**E**,**F**) Sections from AmoD + Pyrv group showing a marked reduction in collagen expression (black arrows). Quantitative analysis of collagen expression including (**G**) distribution area percentage and (**H**) optical density percentage. Data are presented as mean ± SEM (*n* = 6 rats). Statistical analysis was conducted using one-way ANOVA followed by Tukey–Kramer multiple comparison test. * *p* < 0.05, ** *p* < 0.01, *** *p* < 0.001 and **** *p* < 0.0001.

**Figure 6:**
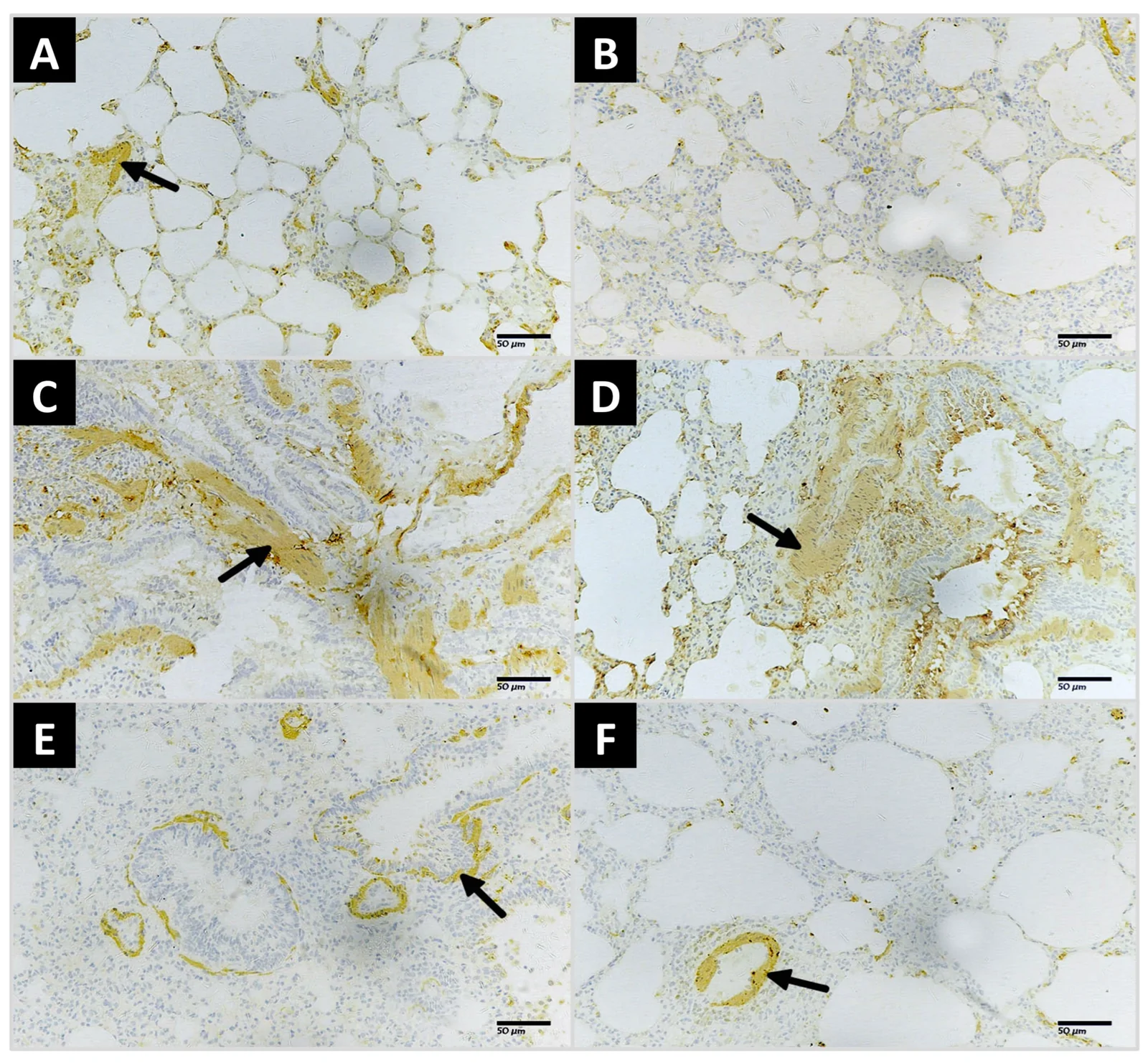

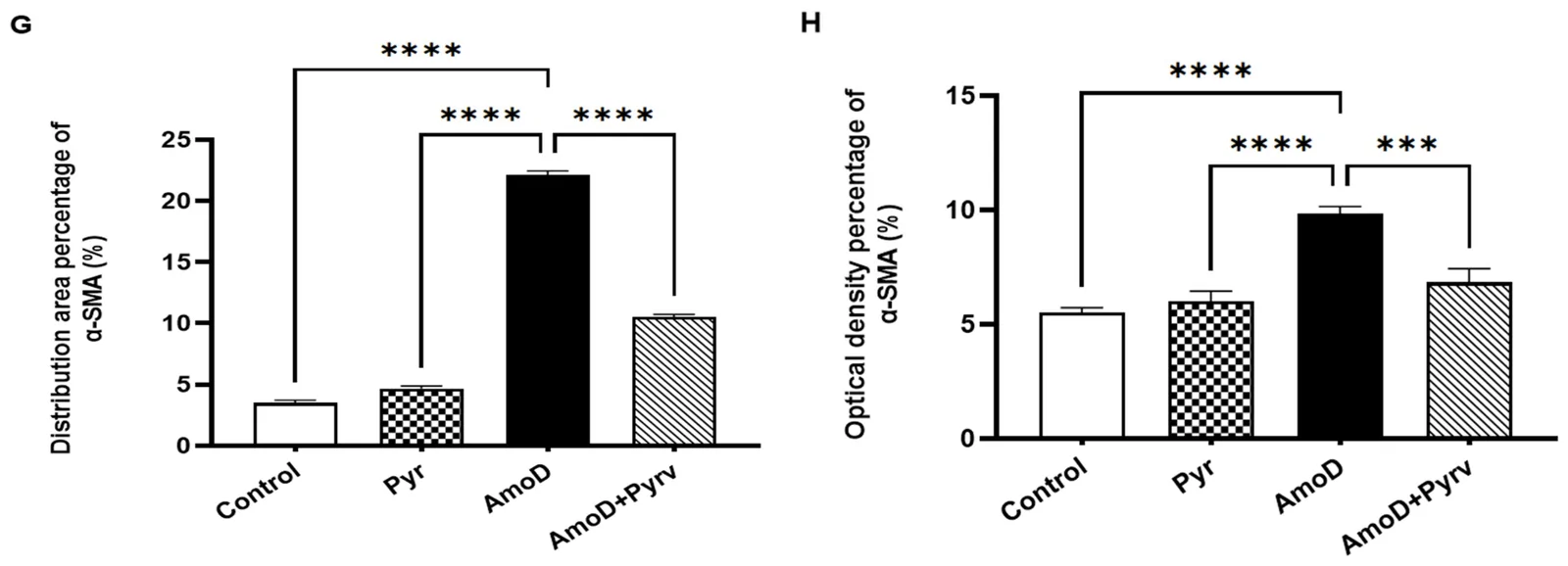
Representative photomicrographs of lung tissue immunohistochemically stained for α-smooth muscle actin (α-SMA), showing expression in alveolar walls and interalveolar septa (200X; scale bar = 50 μm). (**A**) and (**B**) Lung sections from normal control and pyrvinium (Pyrv) control groups, respectively showing weak α-SMA expression. (**C**,**D**) Sections from amiodarone (AmoD)-intoxicated rats exhibiting marked upregulation of α-SMA expression (black arrows). (**E**,**F**) Sections from AmoD + Pyrv group showing a marked reduction in α-SMA expression (black arrows). Quantitative analysis of α-SMA expression including (**G**) distribution area percentage and (**H**) optical density percentage. Data are presented as mean ± SEM (*n* = 6 rats). Statistical analysis was conducted using one-way ANOVA followed by Tukey–Kramer multiple comparison test. *** *p* < 0.001 and **** *p* < 0.0001.

**Figure 7:**
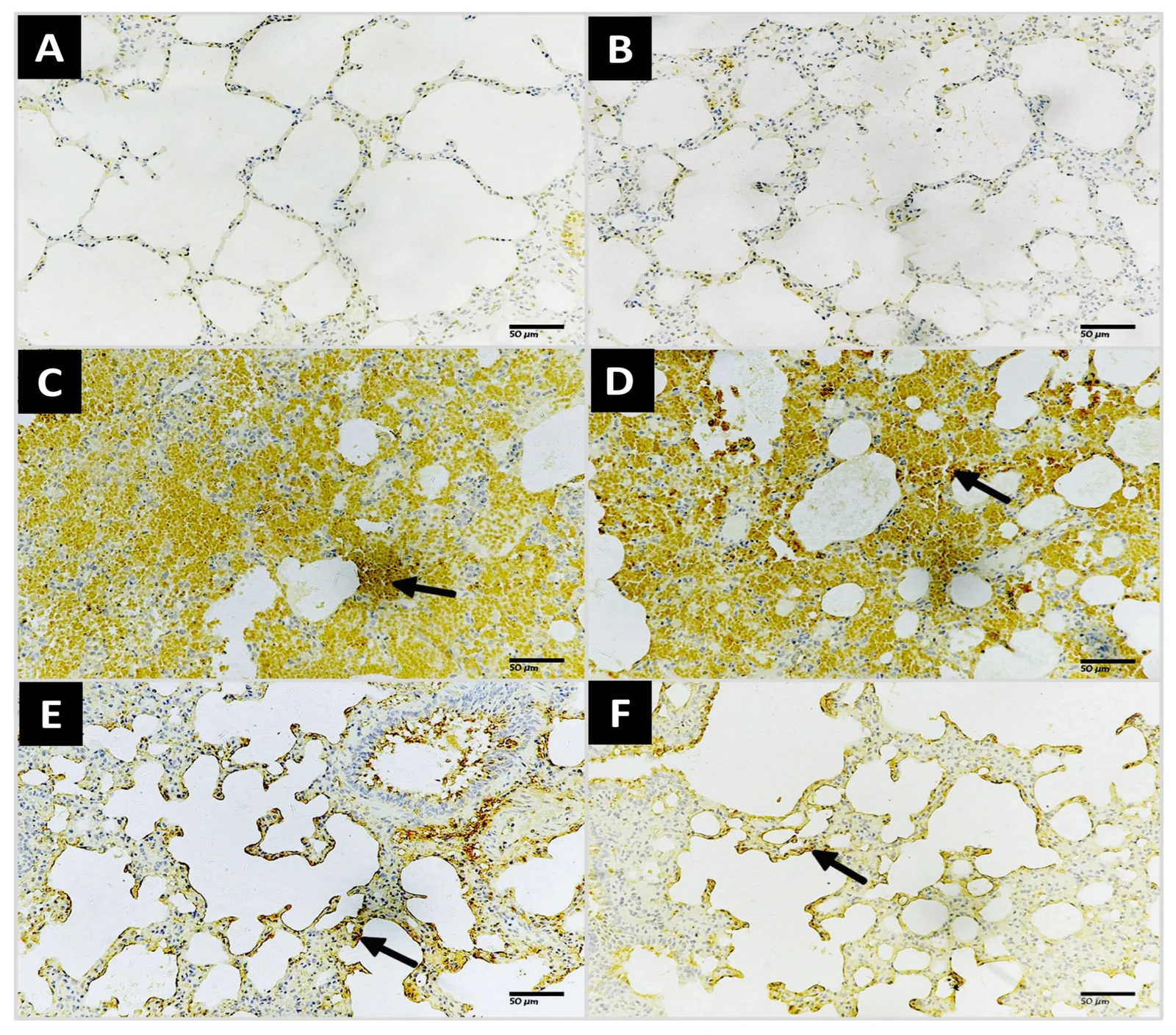

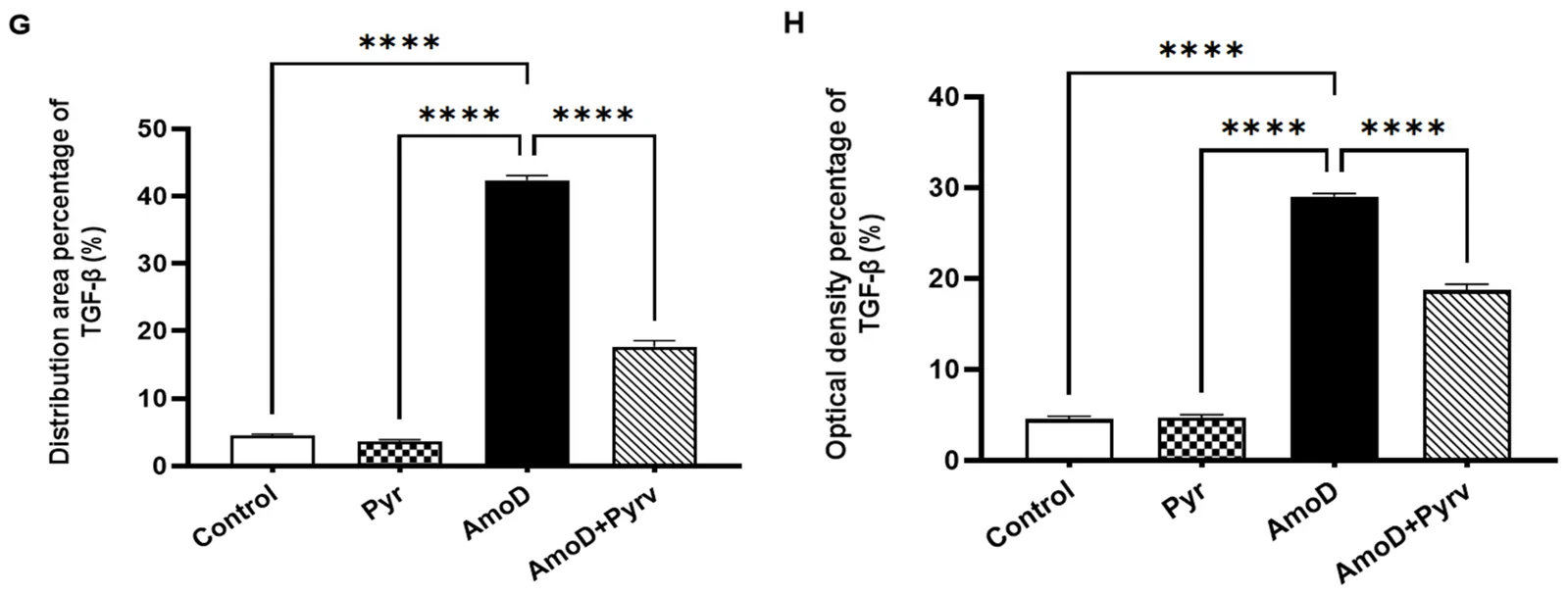
Representative photomicrographs of lung tissue immunohistochemically stained for transforming growth factor-β1 (TGF-β1), showing expression in alveolar walls and interalveolar septa (200X; scale bar = 50 μm). (**A**) and (**B**) Lung sections from normal control and pyrvinium (Pyrv) control groups, respectively showing similarly weak immunoreactivity for TGF-β1. (**C**,**D**) Sections from amiodarone (AmoD)-intoxicated rats exhibiting marked upregulation of TGF-β1 expression (black arrows). (**E**,**F**) AmoD + Pyrv group showing a marked reduction in TGF-β1 expression (black arrows). Quantitative analysis of TGF-β1 expression including (**G**) distribution area percentage and (**H**) optical density percentage. Data are presented as mean ± SEM (*n* = 6 rats). Statistical analysis was conducted using one-way ANOVA followed by Tukey–Kramer multiple comparison test. **** *p* < 0.0001.

Immunohistochemical analysis demonstrated group- dependent differences in pulmonary collagen I, α-SMA, and TGF-β1 expression (Figure 5, Figure 6 and Figure 7). Lung sections from the normal control group exhibited weak immunoreactivity for collagen I, α-SMA, and TGF-β1, representing normal baseline levels of myofibroblast and cytokine activation (Figure 5A, Figure 6A and Figure 7A). Similarly, the Pyrv-treated control group showed weak immunoreactivity for these three markers (Figure 5B, Figure 6B and Figure 7B). In contrast, AmoD-intoxicated rats exhibited increased expression of collagen I, α-SMA, and TGF-β1, characterized by strong, widespread immunopositivity (Figure 5C,D, Figure 6C,D and Figure 7C,D). Co-treatment with Pyrv substantially reduced the immunoreactivity of the three fibrotic markers compared with the AmoD group (Figure 5E,F, Figure 6E,F and Figure 7E,F), confirming that Pyrv mitigates fibrotic remodeling. Quantitative analysis revealed a significant increase in the distribution area and optical density of collagen I (*p* < 0.0001 and *p* = 0.0022, respectively), α-SMA (*p* < 0.0001), and TGF-β1 (*p* < 0.0001) in AmoD-intoxicated rats compared with the control group, while co-treatment with Pyrv significantly alleviates these increases (Figure 5G,H, Figure 6G,H and Figure 7G,H). Together with the Masson’s trichrome findings, these results support that Pyrv attenuates AmoD-associated fibrotic remodeling. The accompanying changes in Wnt/β-catenin-associated proteins suggest a relationship between modulation of this pathway and the observed antifibrotic phenotype.

### 3.4. Pyrv Modulates AmoD-Associated Th1/Th2-Related Cytokine Response in Lung Homogenate

**Figure 8:**
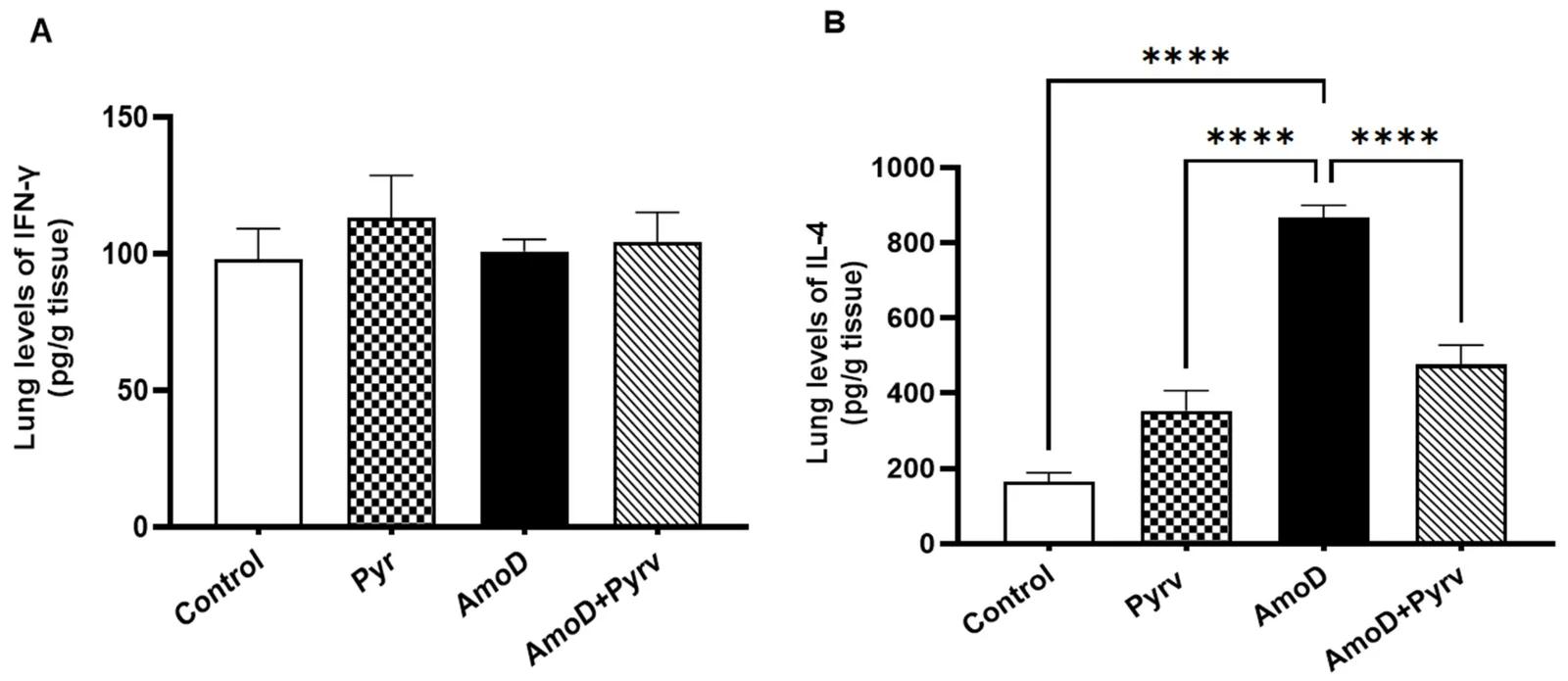
Effect of pyrvinium (Pyrv) on Th1/Th2 cytokine levels in amiodarone (AmoD)-induced lung fibrosis. (**A**) Interferon-γ (IFN-γ) and (**B**) interleukin-4 (IL-4) concentrations measured in lung tissue. Data are presented as mean ± SEM *(n* = 6). Statistical analysis was performed using one-way ANOVA followed by Tukey–Kramer multiple comparison test. **** *p* < 0.0001.

To evaluate the effects of Pyrv on Th1/Th2-related cytokine responses in AmoD-induced lung toxicity, lung levels of IFN-γ (the principal cytokine of activated Th1 cells) and IL-4 (the main cytokine of activated Th2 cells) were measured (Figure 8). The AmoD group exhibited marked immune alterations characterized by a significant increase in IL-4 levels compared with the normal control group (*p* ˂ 0.0001; Figure 8B). In contrast, IFN-γ levels showed a downward trend that did not reach statistical significance (Figure 8A). Therefore, the cytokine pattern observed in the AmoD group is more appropriately described as a Th2-skewed cytokine profile, primarily driven by elevated IL-4. Co-administration of Pyrv significantly reduced IL-4 levels and was accompanied by a trend toward higher IFN-γ levels. These findings suggest that Pyrv attenuated the Th2-associated cytokine alterations induced by AmoD exposure.

### 3.5. AmoD-Induced Pulmonary Injury and Fibrosis Is Associated with Alteration of the Wnt/β-Catenin-Associated Proteins

**Figure 9:**
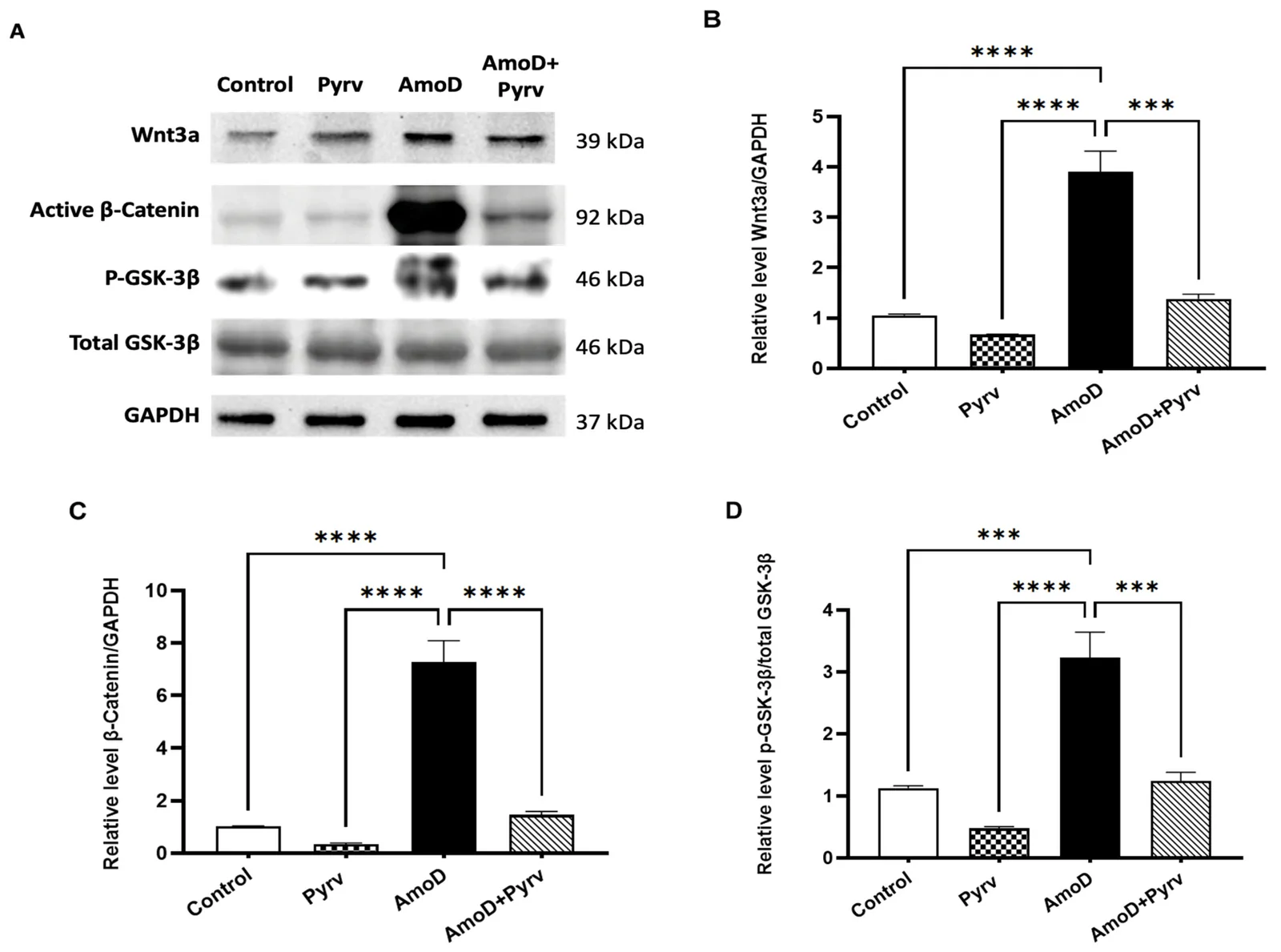
Protein expression levels of Wnt3a, activated β-catenin, ratio of phosphorylated glycogen synthase kinase-3β (p-GSK-3β) to total GSK-3β in lung tissues from normal control, Pyrv, AmoD, and AmoD + Pyrv groups. (**A**) Representative Western Blot images showing the expression of target proteins, with GAPDH used as a loading control. (**B**–**D**) Densitometric analysis of relative protein expression levels of Wnt3a (**B**), activated β-catenin (**C**), and the p-GSK-3β/total GSK-3β ratio (**D**). Protein expression levels were normalized to GAPDH and are presented as mean ± SEM. Statistical analysis was performed using one-way ANOVA followed by Tukey–Kramer multiple comparison test. *** *p* < 0.001 and **** *p* < 0.0001.

To determine whether Wnt/β-catenin-associated proteins were altered during AmoD exposure and whether Pyrv modulated this pathway, protein expression of Wnt3a, active (non-phospho) β-catenin, total GSK-3β, and p-GSK-3 was evaluated by Western Blot (Figure 9). Compared with the normal control group, AmoD-treated rats showed significant increases in Wnt3a (*p* < 0.0001), active β-catenin (*p* < 0.0001), and the p-GSK-3β/total GSK-3β ratio (*p* = 0.0006). Co-administration of Pyrv significantly attenuated the AmoD-induced increases in Wnt3a (*p* = 0.0001), active β-catenin (*p* < 0.0001), and the p-GSK-3β/total GSK-3β ratio (*p* = 0.0009). These findings indicate that AmoD-induced pulmonary injury and fibrosis are accompanied by alterations in Wnt/β-catenin-associated proteins, including increased Wnt3a, active β-catenin, and p-GSK-3β/total GSK-3β ratio. Pyrv treatment was associated with attenuation of these molecular changes. The uncropped full-length immunoblot membranes can be found in Supplementary Material S2.

## 4. Discussion

AmoD-induced pulmonary toxicity is a life-threatening adverse effect that may progress from pulmonary injury and inflammation to irreversible fibrosis [4]. Its pathogenesis is complex and involves direct cellular injury, oxidative stress, immune dysregulation, mitochondrial dysfunction, inflammatory signaling, and phospholipidosis [5,6,7,8]. However, the molecular pathways that contribute to AmoD-induced fibrogenesis remain poorly understood. The Wnt/β-catenin signaling pathway, a key regulator of cell proliferation, differentiation, and tissue repair, has been implicated in the pathogenesis of lung injury [26]; however, its contribution to AmoD-induced lung toxicity has not been previously investigated. The present study investigated the association between Wnt/β-catenin signaling and AmoD-induced pulmonary fibrosis and examined whether pharmacological administration of Pyrv, a Wnt/β-catenin antagonist [18,23], attenuates the biochemical and pathological alterations associated with AmoD exposure.

The present findings demonstrate that AmoD administration produced substantial pulmonary injury, including alveolar septal thickening, hyaline membrane formation, and extensive inflammatory cell infiltration. This result is consistent with previous research in which AmoD caused severe histological injury and pulmonary tissue damage [4,8,27]. Inflammatory cell recruitment may exacerbate this damage through sustained release of pro-inflammatory cytokines such as TNF-α and IL-6, thereby perpetuating a cycle of inflammation and tissue injury. Crucially, Pyrv co-administration markedly preserved alveolar architecture and reduced inflammatory infiltration, indicating its protective effects against AmoD-induced injury. These effects may involve the modulation of the Wnt/β-catenin pathway, which is accompanied by a reduction in inflammatory and injury-related mediators [18,23,28].

The pathological changes induced by AmoD were accompanied by a significant increase in the LW/BW ratio, indicating an increase in relative lung weight. Because the LW/BW ratio is a nonspecific measure, this increase likely reflects the combined effects of pulmonary edema, inflammatory cell infiltration, and fibrotic tissue accumulation rather than true cellular hypertrophy. This interpretation is supported by the concomitant histopathological findings of extensive collagen deposition and increased pulmonary expression of collagen I, α-SMA, and TGF-β1, indicating fibrotic remodeling. These observations are consistent with the established concept that persistent pulmonary injury and inflammation promote fibroblast activation, ECM accumulation, and progressive tissue remodeling [8,13]. In contrast, Pyrv co-administration reduced the LW/BW ratio and substantially attenuated collagen deposition and the expression of collagen I, α-SMA, and TGF-β1, indicating protection against AmoD-induced pulmonary tissue remodeling and fibrosis. As a Wnt/β-catenin antagonist, these findings suggest that AmoD-induced fibrogenesis was associated with the modulation of the Wnt/β-catenin-pathway and that pharmacological modulation of this pathway by Pyrv may mitigate the progression of pulmonary fibrosis.

Consistent with this, pharmacological disruption of Wnt/β-catenin by Pyrv has been shown to attenuate angiotensin II-mediated hypertension-induced cardiac fibrosis by decreasing the expression of α-SMA, fibronectin, and collagen I proteins [28]. In addition, Pyrv (60 μg/kg, p.o.) attenuates non-alcoholic steatohepatitis [29] and cisplatin-induced acute kidney injury [30] and exerts cardioprotective effects in isoproterenol and polymicrobial sepsis models [18,23] via anti-inflammatory and anti-fibrotic mechanisms. Furthermore, it has been reported that Pyrv can block the ability of corneal fibroblasts to upregulate synthesis of α-SMA and collagen I in response to TGF-β1 [19].

The relationship between Wnt/β-catenin signaling and TGF-β1 is particularly relevant because both pathways have been implicated in pulmonary fibrotic remodeling [14,15,16,17]. TGF-β1 is a master driver of pulmonary fibrosis, inducing epithelial-to-mesenchymal transition in alveolar epithelial cells and activating resident fibroblasts into α-SMA-positive myofibroblasts, leading to massive ECM deposition [31,32,33]. In our study, AmoD markedly upregulated TGF-β1 and its downstream effectors α-SMA and collagen I, whereas Pyrv co-treatment suppressed their expression. These data support the concept that β-catenin stabilization is required for full TGF-β1-mediated profibrotic transcription [31,33], and that Pyrv interrupts this synergistic feedback loop.

T helper (Th) cell-associated cytokines play an important role in the regulation of pulmonary fibrosis. Pulmonary fibrogenesis is frequently associated with enhanced expression of Th2-related cytokines, particularly IL-4, which promotes fibroblast activation, ECM deposition, and collagen synthesis, whereas IFN-γ exerts anti-fibrotic effects by limiting fibroblast proliferation and matrix production [34,35]. This phenomenon was also observed in preclinical models of bleomycin-induced pulmonary fibrosis [33,35,36,37]. Consistent with these concepts, AmoD exposure in the present study significantly increased IL-4 levels in lung tissue. Although IFN-γ levels tended to decrease, this change did not reach statistical significance. Accordingly, the observed cytokine alterations are more accurately interpreted as reflecting a Th2-skewed cytokine milieu rather than definitive Th2 cellular polarization. Pyrv treatment significantly attenuated the AmoD-induced increase in IL-4 and was associated with partial restoration of the cytokine profile. These findings suggest that Pyrv may mitigate Th2-associated immune alterations during AmoD-induced pulmonary injury.

An important finding of the present study was the modulation of Wnt/β-catenin-associated proteins in the rats’ lungs following exposure to AmoD, evidenced by significant upregulation of Wnt3a, active (non-phosphorylated) β-catenin, and the p-GSK-3β/total GSK-3β ratio. The observed increase in p-GSK-3β (the phosphorylated, inactive form) reflects a functional impairment of the β-catenin destruction complex. Under baseline conditions, active GSK-3β phosphorylates cytosolic β-catenin, targeting it for ubiquitin-proteasomal degradation. However, the phosphorylation-mediated inactivation of GSK-3β is generally associated with β-catenin stabilization and accumulation promoting its nuclear translocation and transcriptional activity. Our findings align with previous reports demonstrating that fibrotic stimuli induce Ser9 phosphorylation of GSK-3β, thereby facilitating β-catenin stabilization and subsequent myofibroblast differentiation [14]. The present study did not assess the nuclear β-catenin localization or the downstream target genes, which warrant further exploration.

Importantly, Pyrv co-administration attenuated the AmoD-associated increases in Wnt3a active β-catenin, and p-GSK-3β/total GSK-3β ratio. The reduction in GSK-3β phosphorylation reflects increased GSK-3β activity, which may enhance β-catenin degradation and consequently attenuate Wnt/β-catenin signaling. The simultaneous attenuation of Wnt-associated molecular alterations and fibrotic remodeling further supports a relationship between Wnt/β-catenin signaling and the pulmonary response to AmoD. Collectively the present study proposed that AmoD-induced pulmonary injury is associated with fibrotic remodeling, inflammation, and Th2-skewed immune responses, alterations in Wnt/β-catenin-associated proteins. Pyrv attenuates these interconnected alterations, suggesting an association between Wnt/β-catenin-related proteins and AmoD-induced pulmonary injury and fibrosis.

The present study provides a novel discovery and evidence that alterations in Wnt/β-catenin-associated proteins occur in parallel with AmoD-induced lung toxicity and fibrosis. The protective effects observed following Pyrv treatment support the involvement of this pathway and suggest an association between modulation of Wnt/β-catenin-associated proteins and the protective effects of Pyrv. However, an important consideration in our study is that the biological actions of Pyrv cannot be attributed exclusively to modulation of Wnt/β-catenin signaling. Although Pyrv is widely recognized as a pharmacological inhibitor of Wnt signaling through enhancement of β-catenin protein degradation [18,19,23,28,29,30], it is also known to affect other cellular targets and pathways, including casein kinase 1α-dependent signaling, mitochondrial function, cellular metabolism, and cellular stress responses [38]. Therefore, future studies employing complementary mechanistic approaches, such as genetic manipulation of β-catenin or the use of alternative selective Wnt/β-catenin inhibitors, are needed to establish a direct causal role of Wnt/β-catenin activation in AmoD-induced pulmonary fibrosis and to confirm that the protective effects of Pyrv are specifically mediated through modulation of this pathway.

Furthermore, only male rats were included in the study to minimize variability associated with the estrous cycle. However, this limits generalizability and does not address possible sex-dependent responses to AmoD or Pyrv. Future investigations including both male and female animals are warranted to determine whether sex-dependent differences influence disease progression and the therapeutic efficacy of Pyrv.

Another limitation of the present study is the small sample size. Six rats were included in each experimental group based on pilot feasibility data, established paradigms in similar experimental models, and ethical considerations to minimize animal use in accordance with the 3Rs principles (Replacement, Reduction, and Refinement). Although this sample size allowed detection of statistically significant differences for several of the investigated endpoints, no prospective a priori power calculation was performed to determine the minimum sample size based on a predefined effect size, significance level, and statistical power. Future studies should incorporate prospective sample-size calculations based on anticipated effect sizes and variability for the primary outcomes and should include sufficiently powered cohorts to confirm and extend the present findings.

## 5. Conclusions

In conclusion, this study demonstrates for the first time that AmoD-induced pulmonary injury and fibrosis in male rats are accompanied by alterations of canonical Wnt/β-catenin-associated proteins. Pharmacological modulation of Wnt/β-catenin signaling using Pyrv confers significant protection against AmoD-induced lung damage by preserving lung architecture, suppressing pro-inflammatory cytokine expression, attenuating Th2-associated cytokine alterations, and downregulating TGF-β1, α-SMA, and collagen I expression. These findings support the potential involvement of Wnt/β-catenin signaling in AmoD-induced pulmonary fibrosis and that Pyrv’s protective effect was associated with modulation of this pathway. As a future direction, further genetic pathway manipulation or an independent pathway inhibitor is required to establish a direct causal relationship between Wnt/β-catenin activation and AmoD-induced fibrosis and to confirm that Pyrv’s protective effects are specifically mediated through modulation of this pathway. Importantly, because Pyrv is an FDA-approved antiparasitic drug with prior clinical use, it may offer a translational advantage as a repurposed therapeutic candidate for AmoD-induced pulmonary toxicity.

## Figures and Tables

**Table 1:** Lung histopathological scores in different experimental groups.

Groups	Hemorrhage	Edema	Infiltration	Alveolar Wall Thickening	Fibrosis	Total
Normal control	0	0	0	0	0	0
Pyrv	0	0	0	0	0	0
AmoD	3.7 ^a^**** ± 0.17	3.8 ^a^**** ± 0.1	3.78 ^a^**** ± 0.11	3.7 ^a^**** ± 0.16	3.4 ^a^**** ± 0.2	18.6 ^a^**** ± 0.09
AmoD + Pyrv	2.28 ^b^**** ± 0.1	2.5 ^b^**** ± 0.14	3.2 ^b^*** ± 0.14	2.9 ^b^** ± 0.2	1.6 ^b^**** ± 0.2	10.5 ^b^**** ± 0.2

Data are presented as mean ± SEM (*n* = 6 rats). Statistical analysis was conducted using one-way ANOVA followed by Tukey–Kramer multiple comparison test. 0, no injury; 1, <25% injury; 2, 25–50%; 3, 50–75%; and 4, >75%. a: significance versus the normal control group. b: Significance versus AmoD. ** *p* < 0.01, *** *p* < 0.001 and **** *p* < 0.0001.

## Data Availability

All individual animal-level numerical data and image analysis data are available on request from the corresponding author.

## Supplementary Materials

The following supporting information can be downloaded at: https://www.mdpi.com/article/10.3390/pharmaceutics18101289/s1, S1: Statistical data for ELISA, Western Blot, and histological/immunohistochemical quantifications. S2: The uncropped full-length immunoblot membranes.

## Abbreviations

AmoD: Amiodarone; ANOVA: Analysis of variance; ECM: Extracellular matrix; GSK-3β: Glycogen synthase kinase-3β; H&E: Hematoxylin & eosin; IFN-γ: Interferon-γ; IL: Interleukin; Pyrv: Pyrvinium; SEM: Standard error of the mean; TGF-β1: Transforming growth factor-β1; Th: T helper; TNF-α: Tumor necrosis factor-α; Wnt: Wingless/integrase-1; α-SMA: α-smooth muscle actin.
