# Supplementary material for: Pyrvinium Attenuates Amiodarone-Induced Pulmonary Fibrosis in Male Rats: Association with Modulation of Wnt/β-Catenin Related Proteins
Source: Pharmaceutics. 2026 Oct 10;18(10):1289. doi: 10.3390/pharmaceutics18101289 (PMC13708288; doi:10.3390/pharmaceutics18101289)

Wnt3a 39-42 kda (1:1000) SolarBio Cat: K000174M

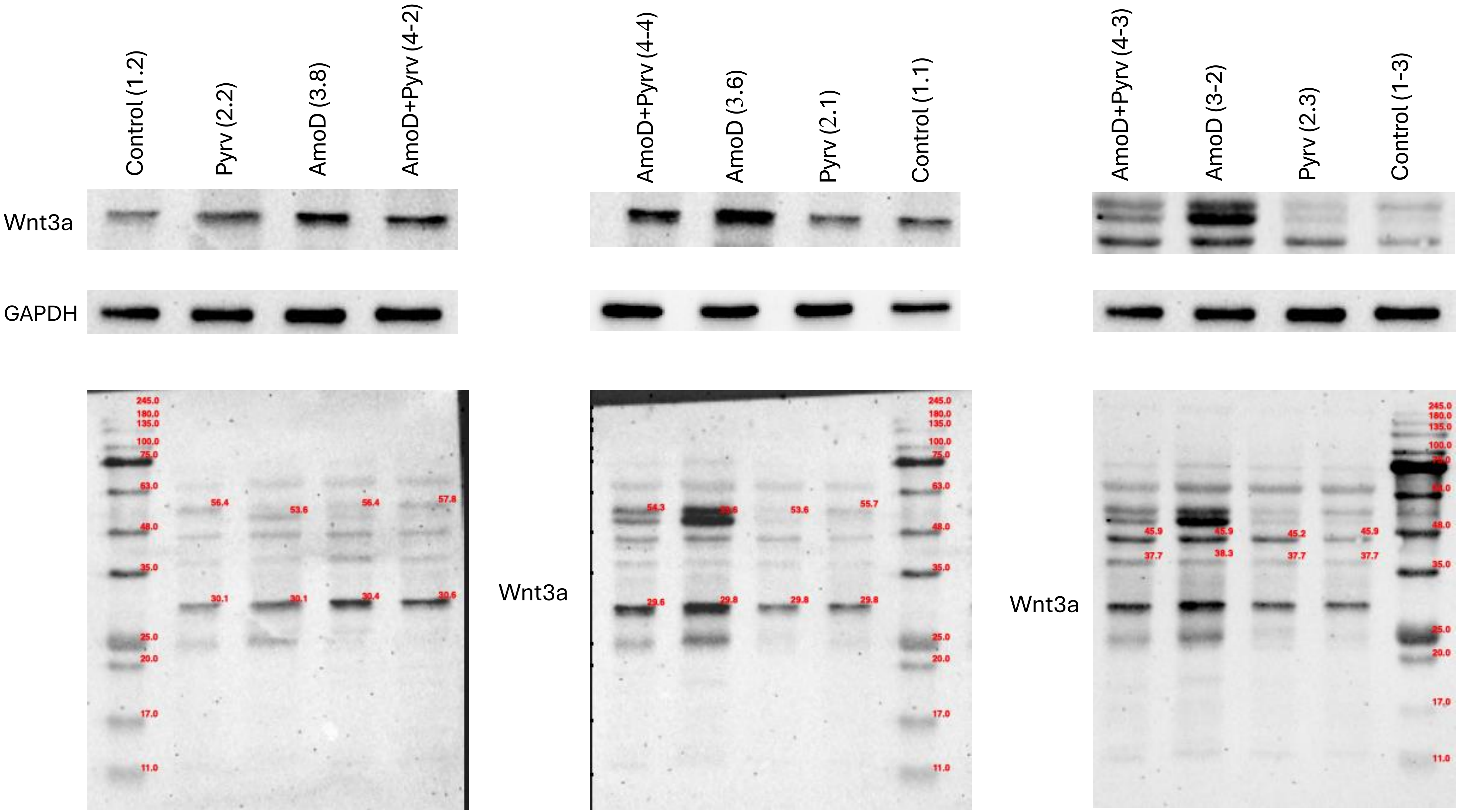

Non-phospho(Active)β-Catenin 92 kda (1:1000) Cell Signaling Cat: 8814

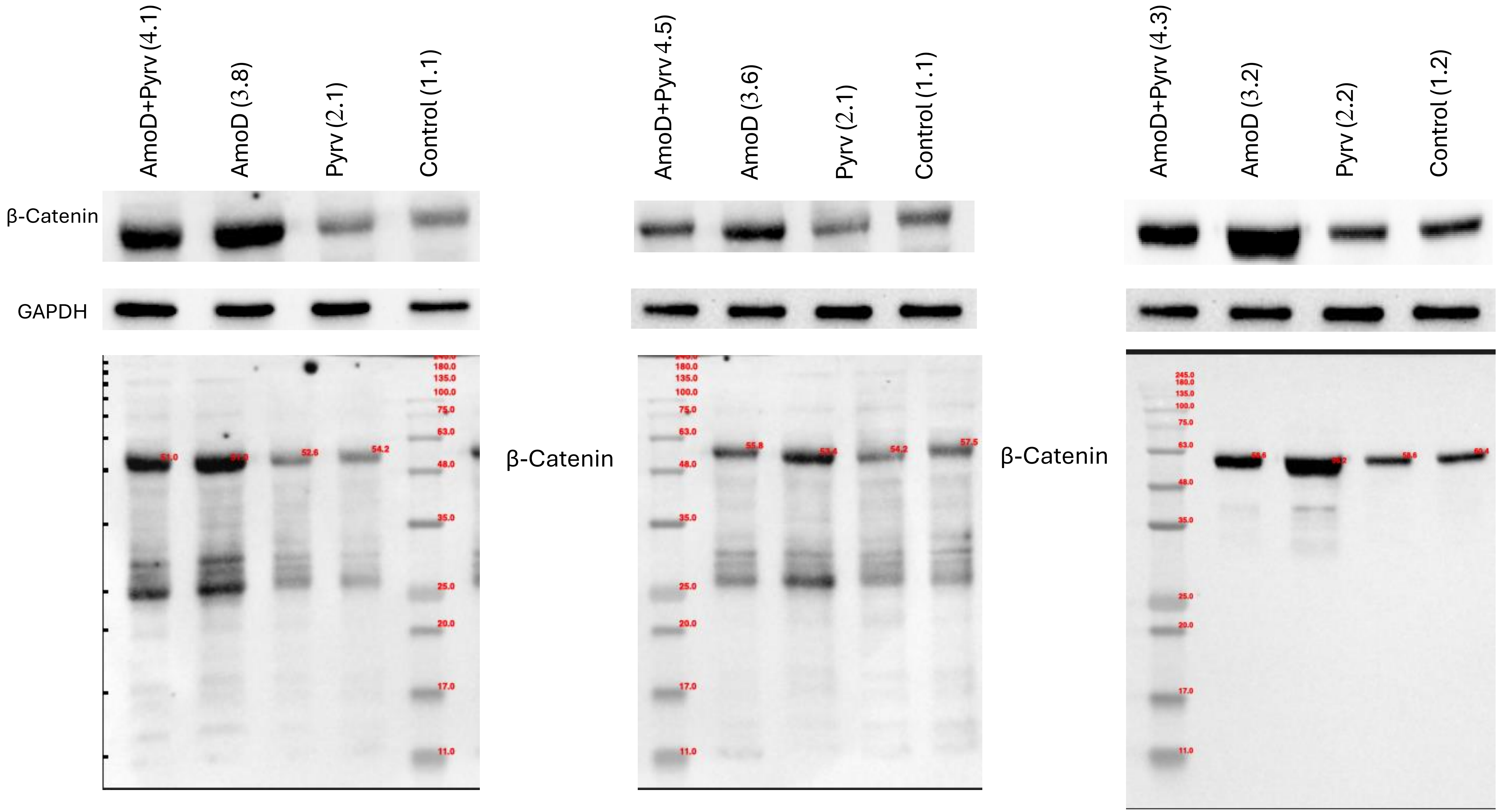

P-GSK3β 46 kda (1:1000) Cell Signaling Cat: #9336

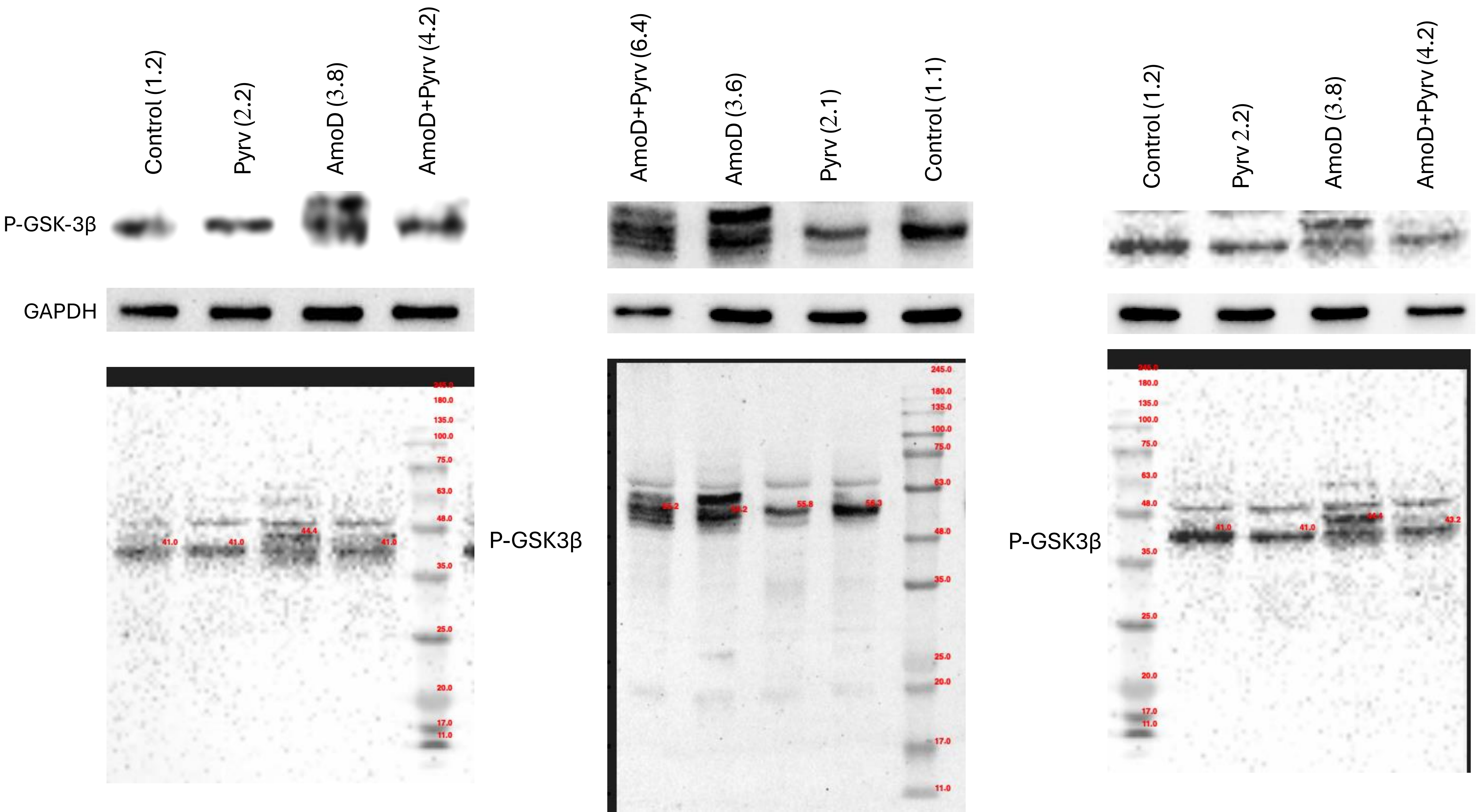

GSK-3β 46 kda (1:1000) ABclonal Cat: A2081

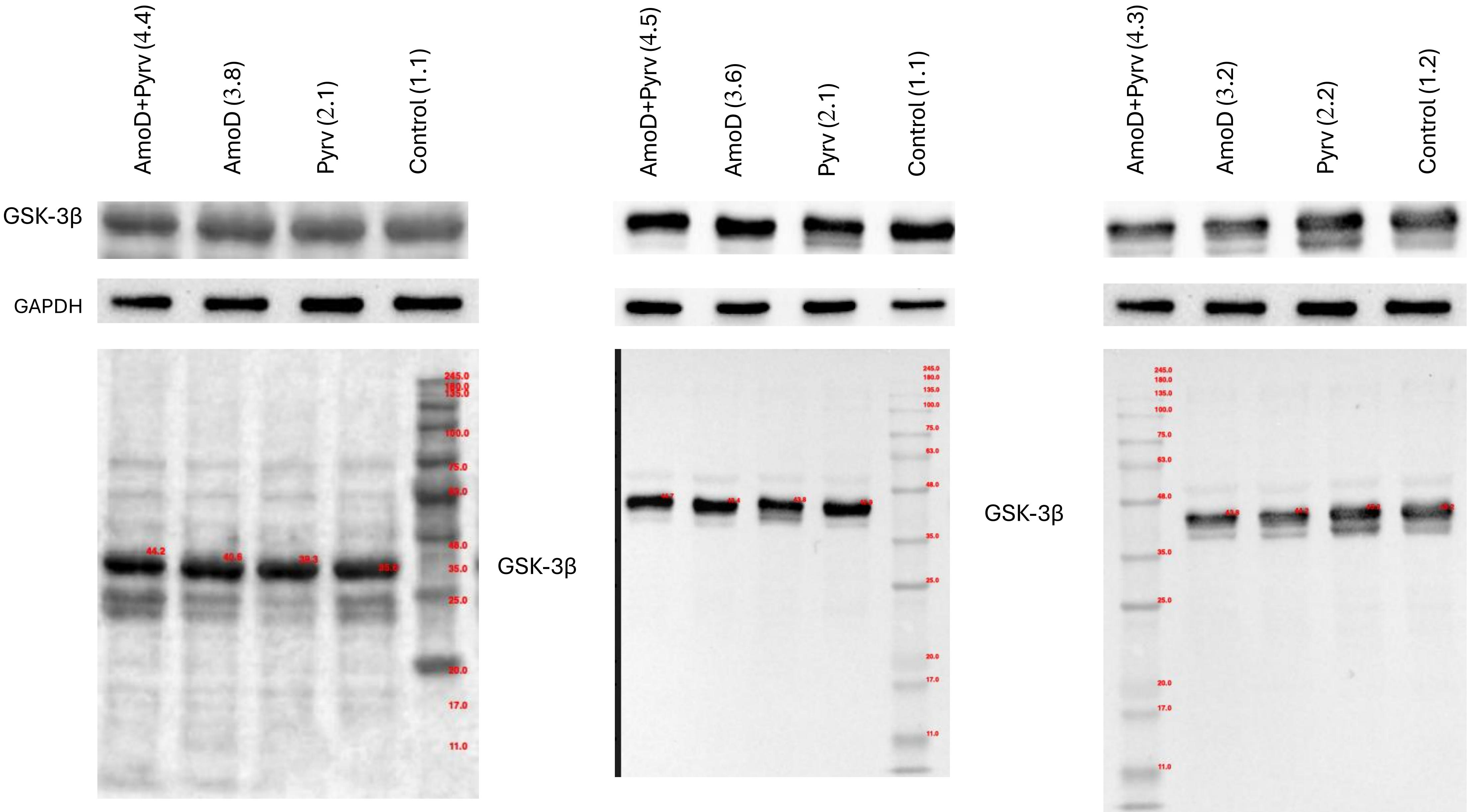

Supplement: Supplementary file 1 [file pharmaceutics-18-01289-s001.zip › S2 (Uncropped full-length immunoblots membranes).pdf]
